# Substituted *N*-(Pyrazin-2-yl)benzenesulfonamides; Synthesis, Anti-Infective Evaluation, Cytotoxicity, and In Silico Studies

**DOI:** 10.3390/molecules25010138

**Published:** 2019-12-29

**Authors:** Ghada Bouz, Martin Juhás, Lluis Pausas Otero, Cristina Paredes de la Red, Ondřej Janďourek, Klára Konečná, Pavla Paterová, Vladimír Kubíček, Jiří Janoušek, Martin Doležal, Jan Zitko

**Affiliations:** 1 Faculty of Pharmacy in Hradec Králové, Charles University, Heyrovského 1203, 50005 Hradec Králové, Czech Republic; juhasm@faf.cuni.cz (M.J.); lluis_pausas@hotmail.com (L.P.O.); cristinaparedesdelared@gmail.com (C.P.d.l.R.); JANDO6AA@faf.cuni.cz (O.J.); konecna@faf.cuni.cz (K.K.); kubicek@faf.cuni.cz (V.K.); janousj2@faf.cuni.cz (J.J.); dolezalm@faf.cuni.cz (M.D.); 2 Department of Clinical Microbiology, University Hospital, Sokolská 581, 500 05 Hradec Králové, Czech Republic; pavla.paterova@fnhk.cz

**Keywords:** anti-infectives, *Mycobacterium tuberculosis*, pyrazinamide, sulfonamide, target fishing

## Abstract

We prepared a series of substituted *N*-(pyrazin-2-yl)benzenesulfonamides as an attempt to investigate the effect of different linkers connecting pyrazine to benzene cores on antimicrobial activity when compared to our previous compounds of amide or retro-amide linker type. Only two compounds, 4-amino-*N*-(pyrazin-2-yl)benzenesulfonamide (MIC = 6.25 μg/mL, 25 μM) and 4-amino-*N*-(6-chloropyrazin-2-yl)benzenesulfonamide (MIC = 6.25 μg/mL, 22 μM) exerted good antitubercular activity against *M. tuberculosis* H37Rv. However, they were excluded from the comparison as they—unlike the other compounds—possessed the pharmacophore for the inhibition of folate pathway, which was proven by docking studies. We performed target fishing, where we identified matrix metalloproteinase-8 as a promising target for our title compounds that is worth future exploration.

## 1. Introduction

**Figure 1:**
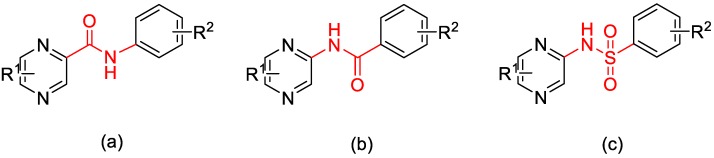
Design rationale: general structure of (**a**) pyrazinecarboxamides = amides; (**b**) *N*-pyrazinylbenzamides = retro-amides; and (**c**) title compounds = sulfonamides.

Despite the availability of an effective treatment regimen against tuberculosis (TB), this infection remains the leading cause of death from infectious diseases worldwide [1]. Inaccessibility to treatment, poor adherence to the complex and lengthy treatment regimen, co-infection with HIV, and drug resistance all contribute to the lethality of this infection [1]. As a part of our long-term research on the derivatives of pyrazinamide (a first-line antitubercular) as potential antimycobacterial agents, we report the synthesis and anti-infective evaluation of a series of *N*-(pyrazin-2-yl)benzenesulfonamides (Figure 1c). Some of our previously prepared pyrazinecarboxamides exerted promising antimycobacterial activity (Figure 1a) [2,3]. As a follow-up, we are investigating different linkers connecting the pyrazine core to the aryl fragment and their effect on in vitro anti-infective activity. In the current series, we aim to study the influence of the isosteric replacement of the retro-amide moiety in our previously published series of *N*-(pyrazin-2-yl)benzamides (Figure 1b) [4] by the sulfonamide moiety in title compounds. The detailed activity comparison between pyrazinecarboxamides (Figure 1a) and *N*-(pyrazin-2-yl)benzamides (Figure 1b) is mentioned elsewhere [4].

**Figure 2:**
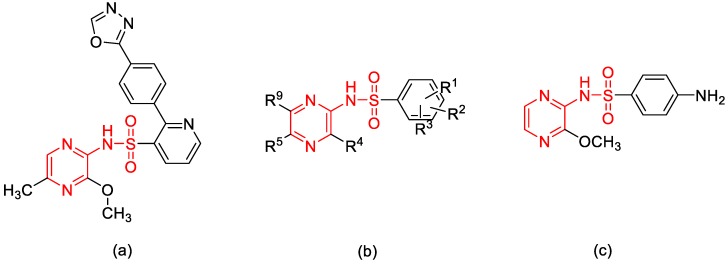
The chemical structure of (**a**) zibotentan, (**b**) halogenated *N*-(pyrazinyl)benzensulfonamides, and (**c**) sulfamethoxypyrazine (sulfalene).

Generally, sulfonamides exert a wide range of biological activities, including anti-tumoral [5], anti-inflammatory [6], anti-convulsant [7], and anti-infective [8], among others. Nevertheless, several compounds containing a pyrazine core directly connected to a sulfonamide moiety—like in our case—are already documented in the literature with a wide range of pharmacological applications [9]. Examples (Figure 2) include zibotentan, an endothelin receptor antagonist with antitumor activity for prostate cancer [10]; halogenated *N*-(pyrazinyl)benzenesulfonamides, which are clathrin-coated pit (CCP) chemokine receptor antagonists used in treating chemokine mediated diseases (such as asthma) [11]; and sulfamethoxypyrazine (sulfalene), which is an antibacterial sulfonamide agent [12]. As for antitubercular activity of sulfonamides, a fixed combination of sulfamethoxazole [4-amino-*N*-(5-methylisoxazol-3-yl)benzenesulfonamide]—trimethoprim [5-(3,4,5-trimethoxybenzyl)pyrimidine-2,4-diamine], also known as co-trimoxazole, has in vitro and in vivo antimycobacterial activity against drug-resistant strains of *Mycobacterium tuberculosis* (*Mtb*), and it is used for such cases [13,14]. Sulfamethoxazole is a known inhibitor of bacterial dihydropteroate synthase (DHPS), while trimethoprim is an inhibitor of dihydrofolate reductase (DHFR) [15,16].

## 2. Results and Discussion

### 2.1. Chemistry

**Scheme 1:**
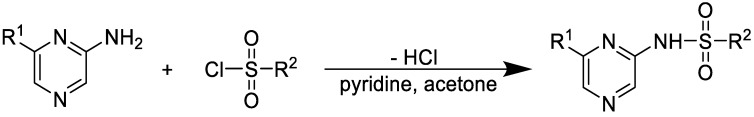
General synthetic reaction. R^1^: H/Cl; R^2^: refer to Table 1.

The chemical synthesis entailed a simple one-step reaction in acetone with pyridine between different sulfonyl chlorides and aminopyrazine (series **1a**–**14a**), or 6-chloroaminopyrazine (series **1b**–**13b**), at room temperature overnight (Scheme 1). Compounds **4a** and **4b** were obtained by reducing compounds **3a** and **3b**, respectively. The purpose of attempting the synthesis with 6-chloroaminopyrazine, using the same sulfonyl chloride was to evaluate the effect of the chlorine atom on anti-infective activity. It is common knowledge that the introduction of a chlorine atom to a molecule increases its lipophilicity. For antimycobacterial activity, the lipophilicity of a compound is an essential factor since mycobacteria have thick, lipid-rich mycolic cell walls [17]. Besides, chloropyrazine derivatives, specifically anilides of 6-chloropyrazine-2-carboxylic acid, were proven previously to possess in vitro antimycobacterial activity [18,19].

The final products were purified using flash chromatography. They were isolated as solid compounds, in yields ranging from 12–70% of chromatographically pure products. Chlorinated compounds (series **1b**–**13b**) had lower yields than the non-chlorinated ones (series **1a**–**14a**) due to the electron-withdrawing property of the chlorine atom that reduces the strength of the primary amine as a nucleophile. The final products were characterized by melting points, ^1^H- and ^13^C-NMR spectra, IR spectroscopy, and elemental analysis. The obtained analytical data fully supported the corresponding proposed structures. In the ^1^H-NMR spectra, the signal of the sulfonamidic hydrogen appeared as a broad singlet at 12.10–10.27 ppm in DMSO-*d_6_*. Pyrazine hydrogens appeared at 8.98–8.04 as two singlets in the case of chlorinated compounds (series **1b**–**13b**), or a singlet and two doublets (*J* = 2.8–2.4 Hz) at 8.40–7.89 ppm in the case of non-chlorinated compounds (series **1a**–**14a**). ^1^H-NMR and ^13^C-NMR spectra of compounds **6a** and **6b** are presented in supplementary materials. Regarding the IR spectra, the final compounds showed signals at 3105–2896 cm^−1^ attributed to the sulfonamidic N–H stretch, at 1609–1460 cm^−1^ attributed to the aromatic C=C stretch, at 1406–1324 cm^−1^ attributed to the S=O unsymmetrical stretch, and at 1189–1092 cm^−1^ attributed to the S=O symmetrical stretch.

### 2.2. Lipophilicity

**Figure 3:**
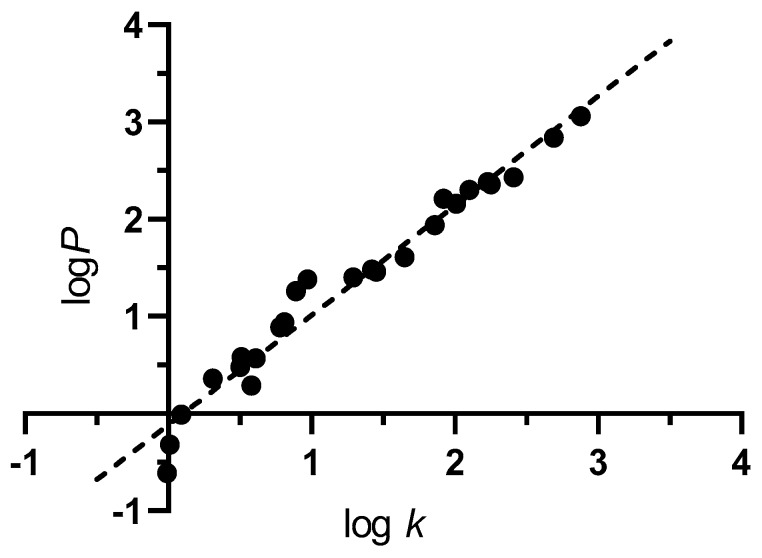
Linear regression of log *P* (calculated) plotted against log *k* (experimental). Equation: log *P* = 1.127 log *k* − 0.1149. (R^2^ = 0.9664, *n* = 25).

Lipophilicity is a significant physico-chemical property in medicinal chemistry and drug design in general. As stated earlier, lipophilicity plays a significant role specifically in the development of new antituberculars. The log *P* values of prepared compounds were calculated using ChemDraw 18.1 (CambridgeSoft, Cambridge, MA, USA). Calculated lipophilicity (log *P*) values were plotted against experimentally determined lipophilicity measures (log *k*). The measures were derived from retention times measured using reverse-phase HPLC. Both values agreed as shown in the graph below (Figure 3).

### 2.3. Acidobasic Properties

The acidic character of sulfonamide moiety determines the ionization state of title compounds at physiological pH and inside the mycobacteria. It further affects lipophilicity and transport across membranes. Three compounds **6b**, **9b** and **13b** were selected and their p*Ka* values were determined experimentally using potentiometric and absorbance methods (Table 2). The values of p*Ka* in water were then predicted using different software to find the best value that gave the closest predictions to the actual experimentally determined values. ChemDraw was not able to predict p*Ka* values, whereas values from the Molecular Operating Environment (MOE, Chemical Computing Group, Montreal, QC Canada)—software is available with a paid license only [21], were far from the experimental ones. Chemicalize was then used for the prediction of p*Ka* values (September 2019, https://chemicalize.com/ developed by ChemAxon http://www.chemaxon.com). The latter values proved to be the closest to the experiments (Table 2), and therefore Chemicalize was selected to predict the p*Ka* values of the remaining compounds (Table 3). The experimental p*Ka* value of compound **4a** was documented in the literature (p*Ka* = 6.04) [22]. This value was close to our predicted value from the Chemicalize web application (p*Ka* = 6.61).

### 2.4. Biological Activity

Six of our title compounds, namely **1a**, **1b**, **2a**, **3a**, **4a**, and **4b**, have been previously mentioned in the literature. Compound **1a** was evaluated as an inhibitor of B-raf kinase [23], compound **1b** was evaluated as a phosphoinositide 3-kinase (PI3K) inhibitor [24], and compound **3a** possessed potent in vitro and in vivo nuclease activity against *Leishmania infantum promastigotes* [25]. Furthermore, compound **4a**—also known as sulfapyrazine—has an antibacterial profile comparable to sulfadiazine [26], a protective effect against sporozoite induced falciparum malaria [27], and sperm production and gonadal development stimulation in animals [28]. Compound **4b**—also known as sulfaclozine—is a commonly used antiprotozoal to treat poultry coccidiosis [29]. It is important to note that compounds **4a** and **4b** were not tested previously against mycobacteria. Sulfaclozine was purchased later as standard. All title compounds were checked for the presence of PAINS and aggregator features using the ZINC15 utility (http://zinc15.docking.org/patterns/home). All the compounds were clear.

#### 2.4.1. Antimycobacterial Activity Evaluation against *Mycobacterium tuberculosis*, *Mycobacterium kansasii*, and *Mycobacterium avium*

**Figure 4:**
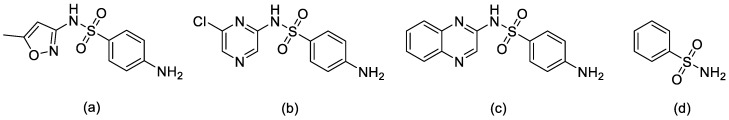
Chemical structures of standards (**a**) sulfamethoxazole, (**b**) sulfaclozine, (**c**) sulfaquinoxaline, and (**d**) benzenesulfonamide.

All synthesized compounds were evaluated for in vitro antimycobacterial activity against *M. tuberculosis* H37Rv, *M. kansasii*, and *M. avium* using microplate alamar blue assay. The antimycobacterial activity results were expressed as a minimum inhibitory concentration (MIC) in μg/mL against isoniazid (INH) as standard. Structurally related sulfonamides, namely, sulfamethoxazole, sulfaclozine, sulfaquinoxaline, and benzenesulfonamide were purchased and subjected to all anti-infective evaluations (Figure 4).

**Figure 5:**
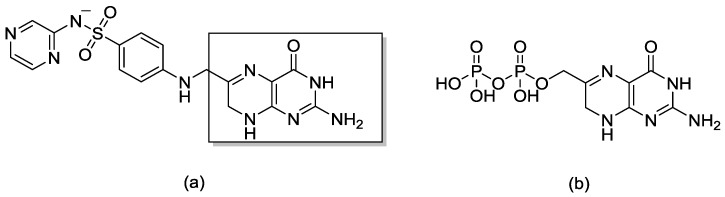
Structures of (**a**) the hypothetic product of compound **4a** and (**b**) 6-hydroxymethyl-7,8-dihydropterin diphosphate (HMDPdiP) used for the docking experiments. Fragment of HMDPdiP in the condensed product is in the rectangle.

Based on the obtained data presented in Table 1, compounds **4a** (MIC*_Mtb_* = 6.25 μg/mL, 25 μM) and **4b** (MIC*_Mtb_* = 6.25 μg/mL, 22 μM) showed good antimycobacterial activity against *Mtb.* None of the other compounds had significant activity against *Mtb.* Compounds **4a** and **4b** are well-known inhibitors of bacterial dihydropteroate synthase (DHPS) [30]. However, their antimycobacterial activity has so far not been investigated. Due to the high similarity of the bacterial and mycobacterial DHPS in the active sites [31], we concluded that the inhibition of *Mtb* growth might be due to the inhibition of mycobacterial DHPS. To rationalize this hypothesis, we used molecular docking to investigate the binding poses of these two compounds in the *Mtb* DHPS (PDB ID: 1eye). Even though mycobacterial DHPS showed only moderate overall amino acid sequence similarity (35 to 39%) to bacterial DHPS [32], the active sites in both DHPSs were highly conserved [31]. Since the only available structure of *Mtb* DHPS (PDB ID: 1eye) misses one of the key conserved flexible loops forming the binding site for a para-aminobenzoic acid (PABA) fragment, as discussed and reviewed further elsewhere [33,34], we docked the hypothetical products of the DHPS-catalyzed condensation of studied compounds **4a** and **4b** with a natural substrate 6-hydroxymethyl-7,8-dihydropterin diphosphate (HMDPdiP, Figure 5) This was done to overcome the inaccuracies that could arise from flexible loop homology modelling. The formation of such a condensed product was described in *Bacillus anthracis* DHPS (PDB ID: 3tye) containing condensed sulfathiazole products [33], where it had also been rationalized using quantum mechanics/molecular mechanics simulations [35]. For more details on the docking experiments, refer to in silico studies from the materials and methods section. With regards to the p*Ka* values of the sulfonamide moiety (Table 2 and Table 3), as well as the proposed mechanism of action (competitive inhibition with DHPS natural substrate—deprotonated PABA), the hypothetical products (Figure 5) of compounds **4a** and **4b** were docked as deprotonated sulfonamides.

**Figure 6:**
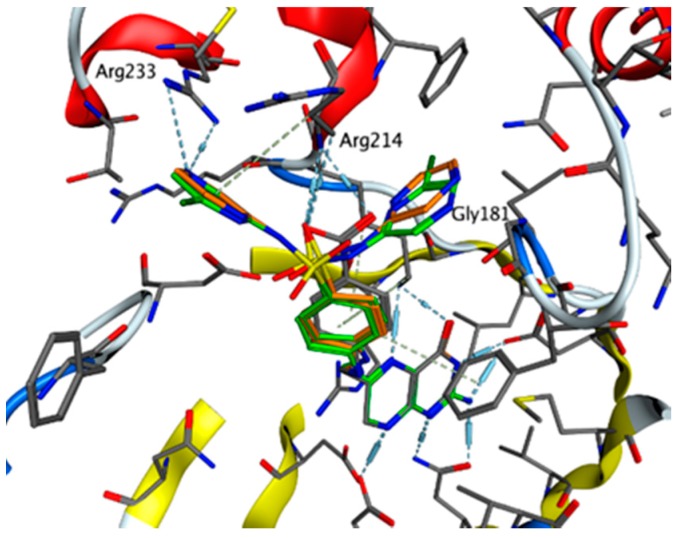
Product of **4a** (orange) and product of **4b** (green) main poses from the docking experiments. The pose of 7,8-dihydropteroate (grey) corresponds to the experimentally determined position seen in bacterial DHPS [e.g., *Burkholderia cenocepacia* DHPS (PDB ID: 2y5s) or *Bacillus anthracis* DHPS (PDB ID: 3tya)].

The docking experiments showed two main potential positions with the poses scoring from −7.9 to −8.3. Positions with the pyrazine core filling the cavity above the active site to the right (formed by Gly181 and Arg214 as seen in Figure 6) had a slightly better score than the positions with the pyrazine to the left. This outcome was probably due to the hydrogen bonds between sulfonamide oxygen and the Arg214 backbone. Similar interactions to Ser221 (corresponding to Arg214 of the mycobacterial DHPS) was also seen in the DHPS of *Bacillus anthracis* for 7,8-dihydropteroate (in this case to the carbonyl oxygen of the carboxylate, PDB ID: 3tya), and it also occurred between the sulfathiazole product (PDB ID: 3tye). On the other hand, poses pointing to the pyrazine to the left had the pyrazine favorably positioned for hydrogen bonding with the guanidine group of Arg233. As seen in Figure 6, substitution of the pyrazine ring with chlorine does not have a significant effect on the predicted binding mode, which justifies the similar antimycobacterial activity of the non-chlorinated compound **4a** (MIC*_Mtb_* = 6.25 μg/mL, 25 μM) to the chlorinated compound **4b** (MIC*_Mtb_* = 6.25 μg/mL, 22 μM) against *Mtb*.

Compounds **2a** (MIC*_M. kansasii_* = 25 μg/mL, 85.5 μM), **2b** (MIC*_M. kansasii_* = 25 μg/mL, 76.5 μM), **11b** (MIC*_M. kansasii_* = 25 μg/mL, 76.3 μM), and **12b** (MIC*_M kansasii_* = 12.5 μg/mL, 36.1 μM), had selective antitubercular activity against *M. kansasii*, and it was higher than INH (MIC of INH was 25 μg/mL, 182.3 μM). For compounds **2a** and **2b**, particularly, this activity was relatable to its chemical structure. The acetamido moiety may have worked as a pro-drug that was later hydrolyzed (supposedly by *M. kansasii* amidase) to the free amino group. This is essential for anti-infective activity based on interference with the folate synthesis pathway [36]. The fact that compounds **2a** and **2b** were inactive against other mycobacterial strains suggested that the hydrolysis machinery in the latter strains was inefficient to liberate the free amino forms due to the difference between substrate specificity of the mycobacterial amidases among different mycobacterial strains [37,38]. It must be noted that the pharmacokinetic profile of compound **4a** in man is already established and it shows that compound **2a** is the major metabolite of compound **4a** [39]. Compounds **4a** (MIC*_M. kansasii_* = 1.56 μg/mL, 6.2 μM) and **4b** (MIC*_M. kansasii_* = 12.5 μg/mL, 44 μM) were also found to be more potent than INH against *M. kansasii*. The addition of the chlorine atom and subsequent increased lipophilicity had a positive effect on antimycobacterial activity against *M. kansasii*, as seen with compounds **11a** (inactive; MIC*_M. kansasii_* > 100 μg/mL, 305.1 μM) vs. **11b** (active; MIC*_M. kansasii_* = 25 μg/mL, 76.3 μM), and **12a** (inactive; MIC*_M. kansasii_* = 100 μg/mL, 321.2 μM) vs. **12b** (active; MIC*_M. kansasii_* = 12.5 μg/mL, 36.1 μM). Compound **4b** (MIC*_M. avium_* = 25 μg/mL, 88 μM) was the only compound to exert antimycobacterial activity against *M. avium*, making it the broadest agent in the spectrum of activity among prepared compounds. Sulfamethoxazole showed potent antimycobacterial activity against *Mtb* (MIC*_Mtb_* = 3.13 μg/mL, 12 μM, less potent than INH), *M. kansasii* (MIC*_M. kansasii_* = 3.13 μg/mL, 12 μM, more potent than INH), and *M. avium* (MIC*_M. avium_* = 12.5 μg/mL, 49 μM, more potent than INH). As for sulfaclozine, its MIC values fully matched that of our freshly prepared compound **4b**, sulfaquinoxaline, which was similar to sulfaclozine in possessing anticoccidiosis activity [40]. It also showed good antimycobacterial activity, while benzenesulfonamide, a classic carbonic anhydrase inhibitor [41], was completely inactive against these three strains.

Estimated p*Ka* values showed no direct correlation with antimycobacterial activity. p*Ka* values of compounds that exhibited antimycobacterial activity were scattered and could not be put in range. Despite having the same p*Ka* value of 7.14, compound **13a** was active against *M. smeg*matis (MIC = 62.5 μg/mL) while compound **1a** was inactive (MIC ≥ 250 μg/mL). The electron-withdrawing property of the additional chlorine atom increased the acidity of the sulfonamidic hydrogen as indicated by lower estimated p*Ka* values, as compared to the corresponding non-chlorinated compounds.

When we sought to compare title compounds to pyrazinecarboxamides (amides) and *N*-pyrazinylbenzamides (retro-amides)—which was our main objective—we found that substitution with the sulfonamide bridge, in general, did not improve the antimycobacterial activity. Compounds **4a** and **4b** were excluded from this comparison as they probably exerted their antimycobacterial activity through a different pathway (folate pathway) than the rest of the title compounds. To achieve a more precise assessment, we compared the antimycobacterial activity against *Mtb* of title sulfonamides to the activity of amides and retro-amides bearing the same substitution both on the pyrazine (R^1^) and benzene (R^2^) ring. It was apparent that the sulfonamide bridge had no positive influence on activity. Some of the antimycobacterial activity against other mycobacterial strains was missing from previous compounds; therefore, we focused on the activity against *Mtb* (Table 4). We note that the methodologies used for MIC determination of amides were not identical to the updated methods used for the retro-amides and title sulfonamides. Therefore, the comparisons should not be taken as a comparison of precise MIC values but as an assessment of general trends instead.

#### 2.4.2. Antimycobacterial Activity Evaluation against *Mycobacterium smegmatis* and *Mycobacterium aurum*

The full series was evaluated for antimycobacterial activity against two fast-growing mycobacterial strains; *M. smegmatis* and *M. aurum*. The latter two strains have the advantage of being avirulent surrogate organisms with better safety profiles and shorter replication time in comparison to *M. tuberculosis* [26]. Microplate alamar blue assay was used for this test against INH, rifampicin, and ciprofloxacin as standards. The most active compounds against *M. smegmatis* were compounds **13a** (MIC*_M. smegmatis_* = 62.5 μg/mL, 219 μM), and interestingly, benzenesulfonamide (MIC*_M. kansasii_* = 31.25 μg/mL, 199 μM), which was inactive against the slow-growing strains of mycobacteria. Some compounds besides sulfaclozine, sulfaquinoxaline, and benzenesulfonamide showed moderate to low activity (MIC*_M. aurum_* = 125–150 μg/mL) against *M. aurum*.

#### 2.4.3. Antibacterial and Antifungal Activity Evaluation

All prepared compounds were screened in vitro for biological activity against four Gram-positive (*Staphylococcus aureus*, methicillin-resistant *Staphylococcus aureus*, *Staphylococcus epidermidis*, *Enterococcus faecalis*) and four Gram-negative (*Escherichia coli*, *Klebsiella pneumoniae*, *Serratia marcescens*, *Pseudomonas aeruginosa*) bacterial strains and eight fungal strains (*Candida albicans*, *Candida krusei*, *Candida parapsilosis*, *Candida tropicalis*, *Aspergillus fumigatus*, *Aspergillus flavus*, *Absidia corymbifera*, *Trichophyton interdigitale*) using microplate dilution assay with MICs determined by the naked eye. No antibacterial or antifungal activities were observed for any of the tested compounds up to the highest tested concentration (500 μM). Compounds **4a** and **4b** satisfied the sulfonamide structural requirements for antibacterial activity [36], yet in our testing, they exerted solely antimycobacterial activity. These findings were consistent with the standard used for screening. Sulfamethoxazole had antimycobacterial activity, but it was not active against any of the tested bacterial strains. The antibacterial activity of the mentioned sulfonamides might be present at concentrations beyond the concentration scale (500–0.97 μM) that we used for such evaluations. In general, the single use of sulfamethoxazole as a quality standard is discouraged due to the low or absence of activity. If it is to be used, then it shall be combined with trimethoprim [42].

#### 2.4.4. In Vitro Cytotoxicity Assays

**Figure 7:**
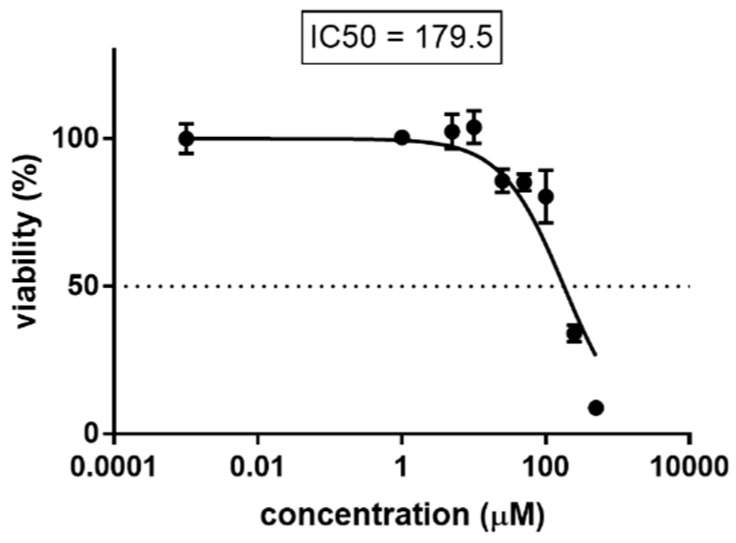
Cytotoxic effect of different incubation concentrations of compound **9b** on HepG2 cells.

Since zibotentan is a pyrazine containing sulfonamide with cytotoxic activity, our compounds were evaluated for similar activity in standard HepG2 (hepatocellular carcinoma) cell line. Furthermore, established antituberculars are known to carry a risk of hepatotoxicity; thus, such evaluations provide an insight into the hepatotoxic effect of the intended compound. Obtained results were expressed by the inhibitory concentration required to decrease the viability of the cell population to 50% (IC_50_) compared to a control of 100% cell viability (Table 1). The used CellTiter 96 assay was based on the reduction of tetrazolium dye MTS in living cells to formazan, which was then determined colorimetrically. Based on the obtained results, none of the prepared compounds exerted significant in vitro cytotoxicity (IC_50_ > 100 µM). Compounds with the chlorine atom (series **1b**–**13b**) were usually more cytotoxic than their corresponding non-chlorinated homologs (series **1a**–**14a**). The most toxic compound among all series was compound **9b** (IC_50_ = 179.5 µM) (Figure 7). When compared to amides and retro-amides, sulfonamides exerted a lesser extent of in vitro cytotoxicity [2,3,4].

#### 2.4.5. Exploring Potential Targets and Activities using Docking Studies

Although designed as potential antimycobacterial compounds, the sulfonamides presented in this paper only exerted low antimycobacterial activity (with the exception of **4a** and **4b**, as discussed above). To discover new potential uses for our compounds, we ran a target fishing campaign based on structural similarity between our *N*-(pyrazin-2-yl)benzensulfonamides and ligands from the RCSB PDB database. First, potential targets were searched in the RCSB database, and then the title compounds were docked into the selected enzymes, after which poses of the molecules were investigated to establish their possible activities. For details of the target fishing and docking procedures, refer to in silico studies from the materials and methods section.

**Figure 8:**
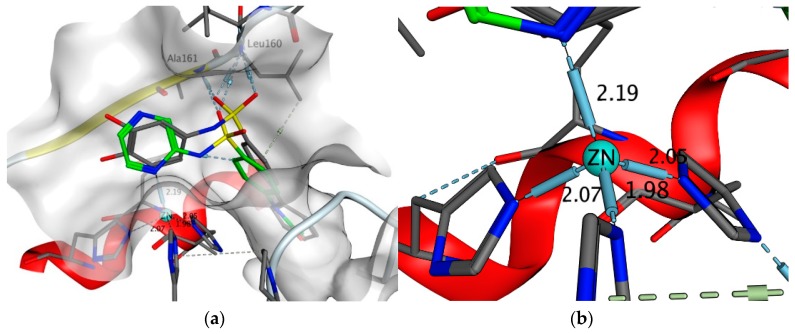
(**a**) Compound **2a** (green carbons) docked to MMP-8 (PDB ID: 5h8x) in comparison to the co-crystalized pose of the original catechol ligand (grey carbons). (**b**) Detail presenting interactions between pyrazine nitrogen N1′ and Zn^2+^ ion coordinated by His residues. (Measurements are in Å).

The search yielded six targets, as presented in Table S1 in supplementary materials. Among these was DHPS, which we have already investigated when determining the mechanism of action of compounds **4a** and **4b**. Then we selected three targets from the remaining five to explore further. Compound **1a** and its derivatives have already been patented as potential inhibitors of B-raf kinase [23]. We then docked our compound into GTPase KRas, with no interesting results. Finally, matrix metalloproteinase-8 (MMP-8; also known as neutrophil collagenase; PDB ID: 5h8x), was investigated in more detail, where its recently described inhibitors are of the above-mentioned structural type (Ar-SONH_2_-Ar) [43]. This endopeptidase is a part of a very complex proteolytic MMP enzyme family, where its overexpression was linked to several pathological processes [43]. As several articles have already described the coordination of metals by pyrazines [44,45], we investigated whether the pyrazine in our compounds could coordinate the Zn^2+^ ion in the MMP-8. This could promote potential inhibitory activity on MMP-8, as presented by Tauro et al. [43]. We tested this hypothesis using molecular docking studies. The results showed that in compounds **2a** (Figure 8) and **12a** (not presented), one of the pyrazine nitrogens was favorably positioned above the Zn^2+^ ion, at a distance of 2.19 Å (comparable to the distance of the His-Zn^2+^ coordinate), as we presumed. Thus, it could easily coordinate the Zn^2+^ in the receptor cavity (Figure 8). In the majority of poses, N1′ was interacting with the metal ion, as seen in Figure 8. The interaction of Zn^2+^ and N4′ was observed only in some poses of 12a. This interaction in addition to the interactions of the sulfonamide moiety was identical to the interactions observed in the original catechol ligand (hydrogen bonds with backbones of Ala161 and Leu160). This in turn gave the basis for the presumed inhibitory activity of our ligands and promoted their further evaluation as inhibitors of MMP-8 or other enzymes of the MMP family.

## 3. Materials and Methods

### 3.1. General Information

All chemicals were of reagent or higher grade of purity. They were purchased from Sigma-Aldrich (Steinheim, Germany) unless stated otherwise. The progress of reactions was checked using Merck Silica 60 F_254_ TLC plates (Merck, Darmstadt, Germany) with UV detection at wavelength 254 nm. Microwave-assisted reactions were performed in a CEM Discover microwave reactor with a focused field (CEM Corporation, Matthews, NC, USA) connected to an Explorer 24 autosampler (CEM Corporation). This equipment was running under CEM’s Synergy^TM^ software for setting and monitoring the conditions of reactions. An internal infrared sensor monitored the temperature of the reaction mixture. All obtained products were purified by a preparative flash chromatograph CombiFlash^®^ Rf (Teledyne Isco Inc., Lincoln, NE, USA) or preparative flash chromatograph Puriflash 420 XS (Intechim Chemicals, Montlucon, France). The type of elution was gradient, using a mixture of hexane (LachNer, Neratovice, Czech Republic) and ethyl acetate (Penta, Prague, Czech Republic) as the mobile phase. Silica gel (0.040–0.063 mm, Merck, Darmstadt, Germany) was used as the stationary phase. NMR spectra were recorded on a Varian VNMR S500 (Varian, Palo Alto, CA, USA) at 500 MHz for ^1^H and 125 MHz for ^13^C. Chemical shifts were reported in ppm (δ) and were referred indirectly to tetramethylsilane via a signal of solvent (2.49 for ^1^H and 39.7 for ^13^C in DMSO-*d*_6_). Infrared spectra were recorded by a spectrometer FT-IR Nicolet 6700 (Thermo Scientific, Waltham, MA, USA) using the attenuated total reflectance (ATR) methodology on a germanium crystal. Elemental analysis was performed on a vario MICRO cube element analyzer (Elementar Analysensysteme, Hanau, Germany). All values regarding elemental analyses were given as percentages. Melting points were determined in open capillary on the Stuart SMP30 melting point apparatus (Bibby Scientific Limited, Staffordshire, UK) and were uncorrected. Yields were expressed as percentages of the theoretical yield, and they referred to the isolated products after all purification steps.

### 3.2. Synthesis

#### 3.2.1. Synthesis of Final Compounds, General Procedure

In a 50 mL beaker, aminopyrazine (3 mmol, 285 mg) or 6-chloroaminopyrazine (3 mmol, 388 mg) was dissolved in 1 mL anhydrous pyridine with stirring at room temperature. Pyridine was used to neutralize the hydrochloric acid generated upon reacting the sulfonyl chloride reagent with the corresponding aminopyrazine or 6-chloroaminopyrazine. Corresponding benzensulfonyl chloride (3 mmol) was dissolved in approximately 2 mL of acetone in a 50 mL pear-shaped beaker with stirring at room temperature. Then the dissolved content in pyridine was added dropwise to the dissolved benzensulfonyl chloride in acetone with stirring at room temperature. The system was then sealed with an appropriate stopper and left to react overnight under the same conditions. The next day, the completion of the reaction was checked by TLC in a 3:1 EtOAc/hexane system. The reaction mixture was transferred to a 100 mL beaker and washed with distilled water to minimize any losses. Then the content was acidified with 10% (m/m) HCl drop-wise until a solid precipitate was formed, which represented the non-ionized form of the product. The acidification step was aimed at ionizing the added pyridine, and any unreacted aminopyrazine or 6-chloroaminopyrazine, to form a salt that dissolved in the aqueous layer during the subsequent extraction step. EtOAc was added as little as needed (30 mL) to dissolve the formed solid precipitate. Distilled water was then added (30 mL) and the two phases were mixed vigorously at room temperature and then transferred to a 500 mL separating funnel. The two layers were then allowed to settle and were separated into two 250 mL beakers. The aqueous layer was rewashed with EtOAc (2 × 30 mL). The combined organic layers from all extractions were then washed one last time with distilled water (100 mL) and then with brine (30 mL). The final organic layer was then transferred to a 150 mL beaker (or less based on the obtained overall volume) and stirred with magnesium sulfate (4 mmol, 500 mg) as a desiccant for 10 min at room temperature. Finally, the dispersion was filtrated through cotton and the resulting filtrate was adsorbed to silica gel to perform flash chromatography using gradient elution 0% to 100% EtOAc in hexane with 0.01% acetic acid as the mobile phase modifier.

#### 3.2.2. Compounds **4a** and **4b**

Compounds **3a** and **3b** (1 mmol) were reduced to the corresponding 4-amino-*N*-(pyrazin-2-yl)benzenesulfonamides (**4a** and **4b**, respectively) with SnCl_2_ dihydrate (1.8 g, 8 mmol) in 10 mL of ethanol with stirring under reflux (80 °C, 30 min). The solvent was evaporated under vacuum and the obtained solid was dissolved in EtOAc (10 mL), and then washed with water (2 × 10 mL) and brine (1 × 5 mL). The final organic layer was then transferred to a 50 mL beaker (or less based on the estimated overall volume) and stirred with magnesium sulfate (2 mmol, 250 mg) as a desiccant for 10 min at room temperature. Finally, the dispersion was filtrated with cotton and the filtrate was adsorbed to silica gel to perform flash chromatography using gradient elution 0% to 100% EtOAc in hexane.

### 3.3. Analytical Data of the Prepared Compounds

*N*-(pyrazin-2-yl)benzenesulfonamide (**1a**). White solid. Yield 68%; M.p. 203–205 °C {in the literature 204–208 °C [25]}; IR (ATR-Ge, cm^−1^): 2956 (NH stretch), 1587, 1558, 1532 (arom. stretch), 1345 (S=O unsymmetrical stretch), 1092 (S=O symmetrical stretch); ^1^H-NMR (500 MHz, DMSO-*d_6_*) δ 11.02 (bs, 1H, sulfonamide), 8.12 (s, 1H, pyrazine), 8.04 (d, *J* = 2.8, 2H, pyrazine), 7.47 (d, *J* = 8.5 Hz, 2H, arom.), 7.14 (d, *J* = 8.5 Hz, 2H, arom.), 7.01 (t, *J* = 7.4 Hz, 2H, arom.). ^13^C-NMR (125 MHz, DMSO-*d_6_*) δ 148.3, 142.3, 140.1, 139.1, 135.1, 133.4, 129.4, 127.2. Elemental analysis found: C, 51.33%; H, 3.87%; N, 17.60%; S, 13.98%. Calculated for C_10_H_9_N_3_O_2_S (MW 235.26): C, 51.05%; H, 3.86%; N, 17.86%; S, 13.63%. CAS#7471-20-7.

*N*-(6-chloropyrazin-2-yl)benzenesulfonamide (**1b**). White solid. Yield 41%; M.p. 198–200.2 °C; IR (ATR-Ge, cm^−1^): 2940 (NH stretch), 1570, 1526, 1499 (arom. stretch), 1398 (S=O unsymmetrical stretch), 1163 (S=O symmetrical stretch); ^1^H-NMR (500 MHz, DMSO-*d_6_*) δ 11.96 (bs, 1H, sulfonamide), 8.40 (s, 1H, pyrazine), 8.24 (s, 1H, pyrazine), 8.05–7.89 (m, 2H, arom.), 7.74–7.62 (m, 1H, arom.), 7.66–7.53 (m, 2H, arom.). ^13^C-NMR (125 MHz, DMSO-*d_6_*) δ 147.5, 145.6, 139.4, 137.5, 133.8, 132.6, 129.5, 127.5. Elemental analysis found: C, 44.23%; H, 2.98%; N, 15.37%; S, 11.76%. Calculated for C_10_H_8_ClN_3_O_2_S (MW 269.70): C, 44.53%; H, 2.99%; N, 15.58%; S, 11.89%. CAS#887310-35-2.

*N*-(4-(*N*-(pyrazin-2-yl)sulfamoyl)phenyl)acetamide (**2a**). Beige solid. Yield 84%; M.p. 243.8–245.6 °C; IR (ATR-Ge, cm^−1^): 3105 (NH stretch), 1592, 1558, 1532 (arom. stretch), 1406 (S=O unsymmetrical stretch), 1162 (S=O symmetrical stretch); ^1^H-NMR (500 MHz, DMSO-*d_6_*) δ 11.42 (bs, 1H, sulfonamide), 10.32 (s, 1H, acetylamide), 8.34 (s, 1H, pyrazine), 8.21 (d, *J* = 2.8, 2H, pyrazine), 7.87 (d, *J* = 8.5 Hz, 2H, arom.), 7.74 (d, *J* = 8.5 Hz, 2H, arom.), 2.06 (s, 3H, –CH_3_). ^13^C-NMR (125 MHz, DMSO-*d_6_*) δ 169.2, 148.4, 143.7, 142.3, 138.9, 134.9, 133.5, 128.7, 118.7, 24.3. Elemental analysis found: C, 49.11%; H, 4.05%; N, 18.98%; S, 10.90%. Calculated for C_12_H_12_N_4_O_3_S (MW 292.31): C, 49.31%; H, 4.14%; N, 19.17%, S, 10.97%.

*N*-(4-(*N*-(6-chloropyrazin-2-yl)sulfamoyl)phenyl)acetamide (**2b**). White solid. Yield 65%; M.p. 217.9–219.9 °C; IR (ATR-Ge, cm^−1^): 3043 (NH stretch), 1592, 1527, 1501 (arom. stretch), 1400 (S=O unsymmetrical stretch), 1164 (S=O symmetrical stretch); ^1^H-NMR (500 MHz, DMSO-*d_6_*) δ 11.90 (bs, 1H, sulfonamide), 10.54 (bs, 1H, acetylamide), 8.30 (s, 2H, pyrazine), 7.89 (m, 4H, arom.), 2.07 (s, 3H, –CH_3_).^13^C-NMR (125 MHz, DMSO-*d_6_*) δ 169.4, 147.6, 145.6, 144.0, 137.1, 132.7, 132.5, 129.0, 118.6, 24.3. Elemental analysis found: C, 44.50%; H, 3.41%; N, 17.24%; S, 9.99%. Calculated for C_12_H_11_ClN_4_O_3_S (MW 326.76): C, 44.11%; H, 3.39%; N, 17.15%; S, 9.81%.

4-nitro-*N*-(pyrazin-2-yl)benzenesulfonamide (**3a**). Yield 69%; M.p. 230.8–231.8 °C {in the literature 233–237 °C [25]}; IR (ATR-Ge, cm^−1^): 2990 (NH stretch), 1565, 1544, 1524 (arom. stretch), 1371 (S=O unsymmetrical stretch), 1160 (S=O symmetrical stretch); ^1^H-NMR (500 MHz, DMSO-*d*_6_) δ 12.10 (bs, 1H, sulfonamide), 8.98 (s, 1H, pyrazine), 8.35 (d, *J* = 2.7 Hz, 2H, pyrazine), 7.87 (d, *J* = 9.2 Hz, 2H, arom.), 7.14 (d, *J* = 9.2 Hz, 2H, arom.). ^13^C-NMR (125 MHz, DMSO-*d*_6_) δ 151.2, 147.9, 145.9, 141.4, 134.1, 135.6, 129.4, 124.8. Elemental analysis found: C, 42.80%; H, 2.65%; N, 19.79% S, 11.13%. Calculated for C_10_H_8_N_4_O_4_S (MW 280.26): C, 42.86%; H, 2.88%; N, 19.99%; S, 11.44%.

*N*-(6-chloropyrazin-2-yl)-4-nitrobenzenesulfonamide (**3b**). Yield 57%; M.p. 218.1–219.8 °C; IR (ATR-Ge, cm^−1^): 2899 (NH stretch), 1535, 1514, 1494 (arom. stretch), 1391 (S=O unsymmetrical stretch), 1105 (S=O symmetrical stretch); ^1^H-NMR (500 MHz, DMSO-*d_6_*) δ 11.89 (bs, 1H, sulfonamide), 8.47 (s, 1H, pyrazine), 8.28 (s, 1H, pyrazine), 8.18 (d, *J* = 9.1 Hz, 2H, arom.), 7.89 (d, *J* = 9.1 Hz, 2H, arom.). ^13^C-NMR (125 MHz, DMSO-*d_6_*) δ 156.1, 151.7, 145.7, 144, 135.4, 134.1, 128, 128, 124.5, 124.5. Elemental analysis found: C, 37.88%; H, 2.05%; N, 17.79% S, 10.13%. Calculated for C_10_H_7_ClN_4_O_4_S (MW 314.70): C, 38.17%; H, 2.24%; N, 17.80%; S, 10.19%.

4-amino-*N*-(pyrazin-2-yl)benzenesulfonamide (**4a**). White powder. Yield 87%; M.p. 254–255.8 °C {in the literature 257–259 °C [46]}; IR (ATR-Ge, cm^−1^): 2961 (NH stretch), 1575, 1561, 1520 (arom. stretch), 1354 (S=O unsymmetrical stretch), 1164 (S=O symmetrical stretch); ^1^H-NMR (500 MHz, DMSO-*d_6_*) δ 11.96 (bs, 1H, sulfonamide), 8.62 (s, 1H, pyrazine), 8.14 (d, *J* = 2.7 Hz, 2H, pyrazine), 7.72 (d, *J* = 9.1 Hz, 2H, arom.), 6.52 (d, *J* = 9.1 Hz, 2H, arom.), 5.6 (bs, 2H, amino). ^13^C-NMR (125 MHz, DMSO-*d_6_*) δ 150.1, 147.9, 145.7, 141.9, 139.3, 135.6, 129, 124.7. Elemental analysis found: C, 47.88%; H, 4.01%; N, 22.16% S, 12.62%. Calculated for C_10_H_8_N_4_O_4_S (MW 250.28): C, 47.99%; H, 4.03%; N, 22.39%; S, 12.81%. CAS#116-44-9.

4-amino-*N*-(6-chloropyrazin-2-yl)benzenesulfonamide (**4b**). Light yellow solid. Yield 81%; M.p. 234.7–236.1 °C; IR (ATR-Ge, cm^−1^): 3442, 3365 (NH_2_ stretch), 2929 (NH stretch), 1585, 1564, 1524 (arom. stertch), 1361 (S=O unsymmetrical stretch), 1150 (S=O symmetrical stretch); ^1^H-NMR (500 MHz, DMSO-*d_6_*) δ 11.06 (bs, 1H, sulfonamide), 8.22 (s, 1H, pyrazine), 8.14 (s, 1H, pyrazine), 7.48 (d, *J* = 9.1 Hz, 2H, arom.), 6.89 (d, *J* = 9.1 Hz, 2H, arom.), 6.02 (bs, 2H, amino). ^13^C-NMR (125 MHz, DMSO-*d_6_*) δ 152.1, 148.9, 145.5, 142.4, 139.2, 134.9, 128.4, 123.9. Elemental analysis found: C, 41.88%; H, 3.05%; N, 19.86% S, 11.23%. Calculated for C_10_H_9_ClN_4_O_2_S (MW 284.72): C, 42.19%; H, 3.19%; N, 19.68%; S, 11.26%. CAS#102-65-8.

*N*-(pyrazin-2-yl)-4-(trifluoromethyl)benzenesulfonamide (**5a**). White solid. Yield 41%; M.p. 154.3–155.5 °C; IR (ATR-Ge, cm^−1^): 2926 (NH stretch), 1609, 1590, 1532 (arom. stretch), 1324 (S=O unsymmetrical stretch), 1145 (S=O symmetrical stretch); ^1^H-NMR (500 MHz, DMSO-*d_6_*) δ 10.27 (bs, 1H, sulfonamide), 8.53 (s, 1H, pyrazine), 8.40 (d, *J* = 2.5 Hz, 2H, pyrazine), 8.04 (m, *J* = 7.8, 1.8, 0.9 Hz, 2H, arom.), 7.89 (m, *J* = 8.7, 7.5, 0.7 Hz, 2H, arom.). ^13^C-NMR (125 MHz, DMSO-*d_6_*) δ 148.8, 142.8, 142.5, 140.5, 136.4, 132.3, 131.4, 130.8, 125.5.

*N*-(6-chloropyrazin-2-yl)-4-(trifluoromethyl)benzenesulfonamide (**5b**). Orange solid. Yield 29%; M.p. 125–127.2 °C; IR (ATR-Ge, cm^−1^): 2930 (NH stretch), 1589, 1525, 1495 (arom. stretch), 1397 (S=O unsymmetrical stretch), 1159 (S=O symmetrical stretch); ^1^H-NMR (500 MHz, DMSO-*d_6_*) δ 12.10 (bs, 1H, sulfonamide), 8.31 (s, 1H, pyrazine), 8.26 (s, 1H, pyrazine), 8.18 (d, *J* = 9.2 Hz, 2H, arom.), 7.68 (d, *J* = 9.2 Hz, 2H, arom.). ^13^C-NMR (125 MHz, DMSO-*d_6_*) δ 151.7, 147.3, 145.6, 138.4, 137.6, 132.8 (q, *J* = 31 Hz), 130.4, 121.5 (q, *J* = 272.1 Hz), 121.0 (q, *J* = 3.7 Hz), 118.9 (q, *J* = 4.2 Hz).

4-methoxy-*N*-(pyrazin-2-yl)benzenesulfonamide (**6a**). White solid. Yield 89%; M.p. 231.5–233.9 °C; IR (ATR-Ge, cm^−1^): 2924 (NH stretch), 1596, 1578, 1531 (arom. stretch), 1344 (S=O unsymmetrical stretch), 1158 (S=O symmetrical stretch); ^1^H-NMR (500 MHz, DMSO-*d_6_*) δ 11.30 (bs, 1H, sulfonamide), 8.30 (s, 1H, pyrazine), 8.18 (d, *J* = 2.4 Hz, 2H, pyrazine), 7.78 (d, *J* = 7.8 Hz, 2H, arom.), 7.64 (d, *J* = 7.8 Hz, 2H, arom.), 4.12 (s, 3H, –OCH_3_). ^13^C-NMR (125 MHz, DMSO-*d_6_*) δ 162.9, 148.4, 142.3, 138.9, 134.9, 131.6, 129.6, 114.5, 55.9. Elemental analysis found: C, 51.12%; H, 4.20%; N, 15.98%; S, 12.12%. Calculated for C_11_H_11_N_3_O_3_S (MW 265.29): C, 49.80%; H, 4.18%; N, 15.84%; S, 12.09%.

*N*-(6-chloropyrazin-2-yl)-4-methoxybenzenesulfonamide (**6b**). White solid. Yield 54%; 185.1–186.3 °C; IR (ATR-Ge, cm^−1^): 2994 (NH stretch), 1579, 1524, 1501 (arom. stretch), 1398 (S=O unsymmetrical stretch), 1164 (S=O symmetrical stretch); ^1^H-NMR (500 MHz, DMSO-*d_6_*) δ 11.91 (bs, 1H, sulfonamide), 8.31 (s, 1H, pyrazine), 7.95 (s, 1H, pyrazine), 7.45 (d, *J* = 9.2 Hz, 2H, arom.), 6.98 (d, *J* = 9.2 Hz, 2H, arom.), 3.81–3.79 (m, 3H, -OCH_3_-). ^13^C-NMR (125 MHz, DMSO-*d_6_*) δ 163.2, 147.6, 145.7, 137.1, 132.4, 130.9, 123.0, 114.6, 55.9. Elemental analysis found: C, 44.48%; H, 3.30%; N, 14.14%; S, 10.56%. Calculated for C_11_H_10_ClN_3_O_3_S (MW 299.73): C, 44.08%; H, 3.36%; N, 14.02%; S, 10.70%.

4-bromo-*N*-(6-chloropyrazin-2-yl)benzenesulfonamide (**7b**). Beige solid. Yield 41%; M.p. 176.5–179.3 °C; IR (ATR-Ge, cm^−1^): 2925 (NH stretch), 1574, 1525, 1497 (arom. stretch), 1396 (S=O unsymmetrical stretch), 1153 (S=O symmetrical stretch); ^1^H-NMR (500 MHz, DMSO-*d_6_*) δ 12.07 (bs, 1H, sulfonamide), 8.36 (s, 2H, pyrazine), 7.89 (d, *J* = 8.6 Hz, 2H, arom.), 7.84 (d, *J* = 8.6 Hz, 2H, arom.). ^13^C-NMR (125 MHz, DMSO-*d_6_*) δ 147.3, 145.6, 138.8, 137.6, 132.7, 132.5, 129.6, 127.7. Elemental analysis found: C, 34.56%; H, 2.10%; N, 12.01%; S, 8.96%. Calculated for C_10_H_7_BrClN_3_O_2_S (MW 348.60): C, 34.46%; H, 2.02%; N, 12.05%; S, 9.20%.

3,5-dimethyl-*N*-(pyrazin-2-yl)benzenesulfonamide (**8a**). White solid. Yield 56%; M.p. 184–187 °C; IR (ATR-Ge, cm^−1^): 2958 (NH stretch), 1588, 1530, 1504 (arom. stretch), 1345 (S=O unsymmetrical stretch), 1189 (S=O symmetrical stretch). ^1^H-NMR (500 MHz, DMSO-*d_6_*) δ 11.30 (bs, 1H, sulfonamide), 8.30 (s, 1H, pyrazine), 7.88 (s, 1H, arom.), 7.74 (s, 2H, arom.), 2.34 (s, 6H, –CH_3_). ^13^C-NMR (125 MHz, DMSO-*d_6_*) δ 148.3, 142.4, 140.1, 139.8, 139, 135, 134.8, 124.6, 20.9. Elemental analysis found: C, 55.34%; H, 4.91%; N, 15.66%; S, 12.30%. Calculated for C_12_H_13_N_3_O_2_S (MW 263.32): C, 54.74%; H, 4.98%; N, 15.96%; S, 12.18%.

*N*-(6-chloropyrazin-2-yl)-3,5-dimethylbenzenesulfonamide (**8b**). Dark yellow-brownish solid. Yield 25%; M.p. 148.1–150.1 °C; IR (ATR-Ge, cm^−1^): 2930 (NH stretch), 1573, 1525, 1494 (arom. stretch), 1394 (S=O unsymmetrical stretch), 1159 (S=O symmetrical stretch); ^1^H-NMR (500 MHz, DMSO-*d_6_*) δ 11.82 (bs, 1H, sulfonamide), 8.35 (s, 2H, pyrazine), 7.58 (s, 2H, arom.), 7.35 (s, 1H, arom.), 2.38 (s, 6H, –CH_3_).^13^C-NMR (125 MHz, DMSO-*d_6_*) δ147.5, 145.6, 139.2, 138.9, 137.2, 135.1, 132.8, 125.2, 20.9. Elemental analysis found: C, 48.35%; H, 4.49%; N, 14.15%; S, 10.05%. Calculated for C_12_H_12_ClN_3_O_2_S (MW 297.03): C, 48.41%; H, 4.06%; N, 14.11%; S, 10.77%.

2,4,6-trimethyl-*N*-(pyrazin-2-yl)benzenesulfonamide (**9a**). White solid. Yield 67%; M.p. 200–201.9 °C; IR (ATR-Ge, cm^−1^): 2949 (NH stretch), 1601, 1586, 1530 (arom. stretch), 1337 (S=O unsymmetrical stretch), 1154 (S=O symmetrical stretch); ^1^H-NMR (500 MHz, DMSO-*d_6_*) δ 11.52 (bs, 1H, sulfonamide), 8.22 (s, 1H, pyrazine), 8.08 (d, *J* = 2.5 Hz, 2H, pyrazine), 7.01 (s, 2H, arom.), 2.64 (s, 6H, -CH_3_), 2.22 (s, 3H, -CH_3_).^13^C-NMR (125 MHz, DMSO-*d_6_*) δ 148.9, 142.5, 142.0, 139.4, 138.1, 134.2, 134.1, 131.8, 22.4, 20.6. Elemental analysis found: C, 56.22%; H, 5.42%; N, 14.88%; S, 11.92%. Calculated for C_13_H_15_N_3_O_2_S (MW 277.34): C, 56.30%; H, 5.45%; N, 15.15%; S, 11.56%.

*N*-(6-chloropyrazin-2-yl)-2,4,6-trimethylbenzenesulfonamide (**9b**). Beige solid. Yield 45%; M.p. 179.7–180.2 °C; IR (ATR-Ge, cm^−1^): 2925 (NH stretch), 1566, 1525, 1498 (arom. stretch), 1331 (S=O unsymmetrical stretch), 1186 (S=O symmetrical stretch); ^1^H-NMR (500 MHz, DMSO-*d_6_*) δ 11.95 (bs, 1H, sulfonamide), 8.24 (s, 1H, pyrazine), 8.15 (s, 1H, pyrazine), 7.04 (s, 2H, arom.), 2.66 (s, 6H, –CH_3_), 2.24 (s, 3H, –CH_3_).^13^C-NMR (125 MHz, DMSO-*d_6_*) δ 156.8, 145.1, 144.9, 141.1, 137.2, 135.1, 134.9, 130.4, 22.6, 21.9. Elemental analysis found: C, 49.87%; H, 4.51%; N, 13.11%; S, 9.99%. Calculated for C_13_H_14_ClN_3_O_2_S (MW 311.78): C, 50.08%; H, 4.53%; N, 13.48%; S, 10.28%.

2,3,5,6-tetramethyl-*N*-(pyrazin-2-yl)benzenesulfonamide (**10a**). White solid. Yield 49%; M.p. 221–223.7 °C; IR (ATR-Ge, cm^−1^): 2955 (NH stretch), 1585, 1526, 1500 (arom. stretch), 1362 (S=O unsymmetrical stretch), 1174 (S=O symmetrical stretch); ^1^H-NMR (500 MHz, DMSO-*d_6_*) δ 11.55 (bs, 1H, sulfonamide), 8.18 (s, 1H, pyrazine), 8.09 (d, *J* = 2.5 Hz, 2H, pyrazine), 7.23 (s, 1H, arom.), 2.55 (s, 6H, –CH_3_), 2.21 (s, 6H, –CH_3_). ^13^C-NMR (125 MHz, DMSO-*d_6_*) δ 148.9, 142.1, 138.1, 135.9, 135.7, 135.3, 134.6, 134.0, 20.7, 17.5. Elemental analysis found: C, 57.45%; H, 5.79%; N, 14.08%; S, 10.72%. Calculated for C_14_H_17_N_3_O_2_S (MW 291.37): C, 57.71%; H, 5.88%; N, 14.42%; S, 11.00%.

*N*-(pyrazin-2-yl)-2,3-dihydrobenzo[b][1,4]dioxine-6-sulfonamide (**11a**). Light yellow solid. Yield 17%; M.p. 264–267.1 °C; IR (ATR-Ge, cm^−1^): 2930 (NH stretch), 1591, 1569, 1540 (arom. stretch), 1350 (S=O unsymmetrical stretch), 1161 (S=O symmetrical stretch); ^1^H-NMR (500 MHz, DMSO-*d_6_*) δ 11.41 (bs, 1H, sulfonamide), 8.35 (s, 1H, pyrazine), 8.21 (d, *J* = 2.4 Hz, 2H, pyrazine), 7.44 (s, 1H, arom.), 6.98 (d, *J* = 8.4 Hz, 2H, arom.), 4.33–4.25 (m, 4H, –CH_2_–). ^13^C-NMR (125 MHz, DMSO-*d_6_*) δ 156.2, 153.9, 147.0, 144.5, 137.4, 136.1, 131.9, 117.3, 115.3, 111.9, 64.2. Elemental analysis found: C, 49.47%; H, 3.97%; N, 14.55%; S, 10.87%. Calculated for C_12_H_10_N_3_O_4_S (MW 293.30): C, 49.14%; H, 3.78%; N, 14.33%; S, 10.93%.

*N*-(6-chloropyrazin-2-yl)-2,3-dihydrobenzo[b][1,4]dioxine-6-sulfonamide (**11b**). White solid. Yield 8%; M.p. 244.6–247°C; IR (ATR-Ge, cm^−1^): 2896 (NH stretch), 1580, 1529, 1498 (arom. stretch), 1323 (S=O unsymmetrical stretch), 1162 (S=O symmetrical stretch); ^1^H-NMR (500 MHz, DMSO-*d_6_*) δ 11.81 (bs, 1H, sulfonamide), 8.34 (s, 2H, pyrazine), 8.28 (s, 1H, arom.), 7.41 (d, *J* = 9 Hz, 2H, arom.), 4.42–4.26 (m, 4H, –CH_2_–). ^13^C-NMR (125 MHz, DMSO-*d_6_*) 156.8, 153.9, 147.0, 144.9, 135.1, 134.9, 131.9, 117.3, 115.3, 111.9, 64.2. Elemental analysis found: C, 43.74%; H, 3.01%; N, 12.54%; S, 9.54%. Calculated for C_12_H_10_ClN_3_O_4_S (MW 327.74): C, 43.98%; H, 3.08%; N, 12.82%; S, 9.78%.

*N*-(pyrazin-2-yl)-[1,1′-biphenyl]-4-sulfonamide (**12a**). Light yellow solid. Yield 79%; M.p. 242.2–244.3 °C; IR (ATR-Ge, cm^−1^): 3070 (NH stretch), 1594, 1566, 1531 (arom. stretch), 1345 (S=O unsymmetrical stretch), 1172 (S=O symmetrical stretch); ^1^H-NMR (500 MHz, DMSO-*d_6_*) δ 11.62 (bs, 1H, sulfonamide), 8.40 (s, 1H, pyrazine), 8.24 (d, *J* = 2.5 Hz, 2H, pyrazine), 7.79 (d, *J* = 9.2 Hz, 4H, arom.), 7.65 (d, *J* = 9 Hz, 2H, arom.), 7.39 (t, *J* = 9 Hz, 3H, arom.). ^13^C-NMR (125 MHz, DMSO-*d_6_*) δ 148.3, 144.9, 142.4, 139.1, 138.9, 138.5, 135.1, 129.3, 128.8, 128.0, 127.6, 127.3. Elemental analysis found: C, 61.32%; H, 4.21%; N, 13.01%; S, 10.30%. Calculated for C_16_H_13_N_3_O_2_S (MW 311.36): C, 61.72%; H, 4.21%; N, 13.50%; S, 10.30%.

*N*-(6-chloropyrazin-2-yl)-[1,1′-biphenyl]-4-sulfonamide (**12b**). White solid. Yield 40%; M.p. 229.3–230.3 °C; IR (ATR-Ge, cm^−1^): 2937 (NH stretch), 1596, 1566, 1525 (arom.), 1397 (S=O unsymmetrical stretch), 1159 (S=O symmetrical stretch); ^1^H-NMR (500 MHz, DMSO-*d_6_*) δ 12.01 (bs, 1H, sulfonamide), 8.34 (s, 1H, pyrazine), 8.14 (s, 1H, pyrazine), 7.49 (d, *J* = 9.2 Hz, 4H, arom.), 7.25 (d, *J* = 9 Hz, 2H, arom.), 7.04 (t, *J* = 9 Hz, 3H, arom.). ^13^C-NMR (125 MHz, DMSO-*d_6_*) δ 147.5, 145.7, 145.2, 138.4, 138.2, 137.5, 132.6, 129.3, 128.9, 128.3, 127.6, 127.3. Elemental analysis found: C, 55.74%; H, 3.54%; N, 12.48%; S, 9.41%. Calculated for C_16_H_12_ClN_3_O_2_S (MW 345.80): C, 55.57%; H, 3.50%; N, 12.15%; S, 9.27%.

*N*-(pyrazin-2-yl)naphthalene-2-sulfonamide (**13a**). White solid. Yield 57%; M.p. 223.5–224.7 °C; IR (ATR-Ge, cm^−1^): 2925 (stretch NH), 1589, 1576, 1530 (arom. stretch), 1341 (S=O unsymmetrical stretch), 1160 (S=O symmetrical stretch); ^1^H-NMR (500 MHz, DMSO-*d_6_*) δ 11.67 (bs, 1H, sulfonamide), 8.77 (s, 1H, pyrazine), 8.65 (d, *J* = 2.4 Hz, 2H, pyrazine), 7.92 (m, 3H, arom.), 7.68 (m, 4H, arom.).^13^C-NMR (125 MHz, DMSO-*d_6_*) δ 148.3, 142.3, 139.1, 137.1, 135.1, 134.6, 131.7, 129.6, 129.5, 129.3, 128.8, 128.0, 127.9, 122.4. Elemental analysis found: C, 58.91%; H, 3.89%; N, 14.38%; S, 11.61%. Calculated for C_14_H_11_N_3_O_2_S (MW 285.32): C, 58.94%; H, 3.89%; N, 14.73%; S, 11.24%.

*N*-(6-chloropyrazin-2-yl)naphthalene-2-sulfonamide (**13b**). Light yellow. Yield 33%; M.p. 204.6–207 °C; IR (ATR-Ge, cm^−1^): 3056 (NH stretch), 1522, 1505, 1460 (arom. stretch), 1398 (S=O unsymmetrical stretch), 1169 (S=O symmetrical stretch); ^1^H-NMR (500 MHz, DMSO-*d_6_*) δ 12.05 (bs, 1H, sulfonamide), 8.35 (s, 1H, pyrazine), 8.30 (s, 1H, pyrazine), 8.20 (m, arom.), 7.93 (m, 3H, arom.), 7.76–7.64 (m, 4H, arom.). ^13^C-NMR (125 MHz, DMSO-*d_6_*) δ 156.0, 147.5, 146.0, 145.6, 137.4, 136.3, 134.7, 132.5, 131.6, 130.6, 129.6, 129.6, 128.7, 128.0, 128.0. Elemental analysis found: C, 52.89%; H, 3.10%; N, 13.07%; S, 10.04%. Calculated for C_14_H_10_ClN_3_O_2_S (MW 319.76): C, 52.59%; H, 3.15%; N, 13.14%; S, 10.03%.

*N*-(pyrazin-2-yl)quinoline-6-sulfonamide (**14a**). Beige solid. Yield 61%; M.p. 249.4–252 °C; IR (ATR-Ge, cm^−1^): 2927 (NH stretch), 1585, 1552, 1533 (arom. stretch), 1339 (S=O unsymmetrical stretch), 1159 (S=O symmetrical stretch); ^1^H-NMR (500 MHz, DMSO-*d_6_*) δ 11.53 (bs, 1H, sulfonamide), 8.87 (s, 1H, pyrazine), 8.66 (d, *J* = 2.5 Hz, 2H, pyrazine), 7.99–7.14 (m, 6H, arom.). ^13^C-NMR (125 MHz, DMSO-*d_6_*) 153.1, 151.6, 147.5, 143.2, 137.1, 136.5, 136.0, 135.2, 131.7, 131.6, 128.6, 126.0, 122.8. Elemental analysis found: C, 54.82%; H, 3.37%; N, 19.77%; S, 10.40%. Calculated for C_13_H_10_N_4_O_2_S (MW 286.31): C, 54.54%; H, 3.52%; N, 19.57%; S, 11.20%.

### 3.4. Log k Determination

Log *k* was determined using an Agilent Technologies 1200 SL liquid chromatograph with a Diode-array Detector SL G1315C (Agilent Technologies Inc., Colorado Springs, CO, USA), with pre-column ZORBAX XDB-C18 5 µm, 4 mm × 4 mm and column ZORBAX Eclipse XDB-C18 5 µm, 4.6 mm × 250 mm (both Agilent Technologies Inc.). The mobile phase consisted of MeOH (HPLC grade, 70%) and H_2_O (HPLC-Milli-Q Grade, 30%) with 0.01% acetic acid as the mobile phase modifier. The flow rate was 1.0 mL/min, and samples were injected at a volume of 20 µL, where the column temperature was 30 °C. The detection and monitoring wavelengths were 210 nm and 270 nm, respectively. Retention times (R_t_) were measured in minutes. The dead time of the system (D_t_) was determined as the retention time of the KI methanolic solution. Capacity factors k for individual compounds were calculated according to the formula k = (R_t_ − D_t_)/D_t_. Capacity factor k was converted to the log scale and (log *k*), and it was used as a measure of lipophilicity.

### 3.5. pKa Determination

For the spectrophotometric method, 1 μM solution of the compound in question was prepared in methanol. The spectra of the sample at several (at least seven) different pH values were measured against blank (water) using a Helios spectrophotometer (Unicam Cambridge). The pH valued covered the region in which all sample molecules were converted from ionized to molecular form or vice versa. Each cuvette contained 200 μL of the sample solution and 1.8 mL of the corresponding buffer. The pH values used and the absorbances allowed us to calculate the p*Ka* of the sample according to the following equation:

$$p\mathrm{Ka}=\mathrm{pH}+\log\left(\frac{Ai-A}{A-Au}\right)$$

where *A* is the absorbance of the compound at the corresponding buffer, *Ai* is the absorbance of the ionized state of the compound and *Au* is the absorbance of the unionized state. The final p*Ka* was the average of all the p*Ka* at different pH values. For the potentiometric measurements, 1 μM solution of the compound in question was prepared in 3:2 water:methanol, with the addition of 100 μL of 0.1% NaOH. Then small amounts (100 μL) of 0.01% HCl were added repeatedly and the pH was measured each time using a pH meter (inoLab^®^ pH 7110 Czech Republic). The inflection point of the titration curve determined the p*Ka*. Each compound was measured two times and the resulting p*Ka* was the average of two values.

### 3.6. Biological Assays

For details, refer to the supplementary materials.

### 3.7. In Silico Studies

#### 3.7.1. DHPS Docking

For the docking studies, we used the rigid receptor settings of the template docking feature incorporated in MOE 2019.0101 (Chemical Computing Group, Montreal, Canada) [21]. For the template, all heavy atoms of the pteridine core were selected as these atoms, in reality, were already inside the enzyme. The compounds were docked to the publicly available structure of *Mtb* DHPS (PDB ID: 1eye). Final refinement (Refinement: Rigid) of the pose was done using the default GBVI/WSA dG scoring function. To test the ability of such settings to predict the correct binding poses, we docked 7,8-dihydropteroate using the same settings, and we observed correct interactions of the *p*-aminobenzoic acid carboxylic group. This was as seen in the crystallographic structures of such products in, for example, *Burkholderia cenocepacia* DHPS (PDB ID: 2y5s) or *Bacillus anthracis* DHPS (PDB ID: 3tya).

#### 3.7.2. Target Fishing

For the target fishing, we searched the RCSB PDB database (https://www.rcsb.org/) for ligands containing benzenesulfonamide moiety using the ‘Ligand search’ utility. The obtained ligands were imported into the MOE database (MOE 2019.0101) [21] and then filtered using the SMARTS string “cS(O)(O)[NH]c” to retrieve ligands that met the aromatic-SONH-aromatic scaffold. The resulting database was used for similarity searching based on the MACCS structural keys (bit-packed) with Tanimoto > 0.75, based on compound **1a** as a template. The resulting compounds were inspected visually to remove all unrelated compounds, e.g., compounds with a highly substituted aromatic ring. Ligands in the final selection were searched in the RCSB PDB database for the associated co-crystalized targets, which were considered candidate targets for the title molecules of this manuscript.

#### 3.7.3. Other Targets Docking

For the docking study, we used the MOE 2019.0101 [21]. The docking was carried out on a rigid receptor using default dock settings. The poses generated by the Triangle Matcher placement method were scored using the London dG function. The best poses were refined (Refinement: Rigid) and scored using the GBVI/WSA dG scoring function. Concerning the predicted p*Ka* values of our compounds, the deprotonated forms were used as ligands.

## 4. Conclusions

To conclude, as a part of our ongoing research, we designed and synthesized 25 different substituted *N*-(pyrazin-2-yl)benzenesulfonamides. This effort was an attempt to study the influence of different linkers connecting the pyrazine core to different aromatic moieties on antimicrobial activity and antitubercular activity in particular (amide vs. retro-amide vs. sulfonamide linkers). Nineteen of the title compounds were not previously described in literature when we searched on the 5th of March 2019 using SciFinder (Columbus, OH, USA) and Reaxys. Compounds **1a**, **1b**, **2a**, **3a**, **4a**, and **4b** were published but not evaluated for antimycobacterial activity. We evaluated the prepared compounds for their anti-infective activity against *Mycobacterium tuberculosis* and four other nontubercular mycobacterial strains, along with antibacterial and antifungal activity evaluation. Based on the obtained results, we concluded that the introduction of a sulfonamide linker did not bring significant improvement to antimicrobial activity when compared to the other amide or retro-amide linkers. The exception includes compounds **4a** (MIC*_Mtb_* = 6.25 μg/mL, 25 μM) and **4b** (MIC*_Mtb_* = 6.25 μg/mL, 22 μM), which showed good antitubercular activity against *Mtb.* Such activity may be attributed to the presence of a free amino group in the molecule, which characterizes antimicrobial sulfonamides, and not to the sulfonamide linker itself. Compounds **2a** (MIC*_M. kansasii_* = 25 μg/mL) and 12b (MIC*_M. kansasii_* = 12.5 μg/mL) selectively inhibited the growth of *Mycobacterium kansasii*, suggesting that these two compounds targeted a certain pathway not common among all mycobacterial strains. Compound **13a** showed promising antimycobacterial activity against *M. smegmatis* (MIC*_M. smegmatis_* = 62.5 μg/mL, 219 μM). Some compounds showed moderate to weak activity against *M. aurum*. Among them all, compound **4a** had a broad spectrum of antimycobacterial activity with good activity against *Mtb* (MIC = 6.25 μg/mL) and moderate activity against *M. kansasii* (MIC = 12.5 μg/mL), *M. avium* (MIC = 25 μg/mL), and *M. aurum* (MIC = 250 μg/mL). No antibacterial or antifungal activity was observed for any of the prepared compounds. Regarding in vitro cytotoxicity, when compared to reference amides or retro-amides, the title compounds had a more favorable profile. The title compounds were explored for other activities by docking studies. These studies further rationalized the antimycobacterial activity of the most active compounds **4a** and **4b** as inhibitors of *Mtb* DHPS. Finally, we presented potentially active poses of the compound **2a** that could rationalize its inhibitory activity on MMP-8 or other MMP family enzymes implicated in several pathological conditions.

## Figures and Tables

**Table 1:** Structure of prepared compounds, antimycobacterial activity expressed by minimum inhibitory concentrations (MIC), and HepG2 cytotoxicity expressed by IC_50_ values.

a		b					IC_50_ (µM)
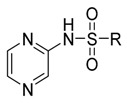		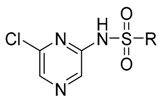					
No.	R (the Cut Line Represents the Attachment Point)	Antimycobacterial Activity MIC (µg/mL)					
		*Mtb* H37Rv	*M. kansasii*	*M. avium*	*M. smeg*	*M. aurum*	
**1a**	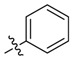	>100	100	>100	≥250	≥500	>1000
**1b**		50	100	>100	250	≥500	731
**2a**	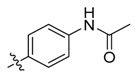	>100	25	>100	≥500	≥500	>500 *
**2b**		>100	25	>100	250	≥500	>500 *
**3a**	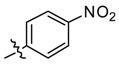	>100	>100	>100	>500	>500	>1000
**3b**		>100	>100	>100	250	125	>1000
**4a**	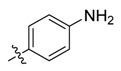	6.25	1.56	100	≥500	250	775.6 *
4b ***		6.25	12.5	25	≥500	250	>500
**5a**	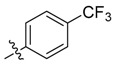	>100	50	>100	250	250	>1000
**5b**		100	>100	>100	250	250	364
**6a**	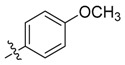	>100	>100	>100	≥500	≥500	>1000
**6b**		>100	100	>100	≥500	≥500	>1000
**7b**	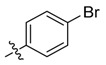	100	50	>100	125	125	308
**8a**	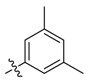	>100	50	>100	≥500	250	>500 *
**8b**		50	>100	50	≥500	≥500	523.3
**9a**	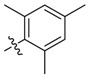	>100	>100	>100	125	≥500	>250 *
**9b**		>100	50	>100	≥500	≥500	179.5
**10a**	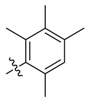	>100	50	>100	≥500	≥500	>250 *
**11a**	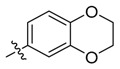	>100	>100	>100	≥500	250	>1000
**11b**		>100	25	>100	≥500	≥500	778.5
**12a**	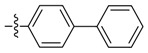	100	100	>100	≥500	≥500	>250 *
**12b**		100	12.5	>100	≥500	≥500	302
**13a**	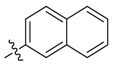	>100	50	>100	62.5	≥500	>500 *
**13b**		50	50	>100	125	250	>1000
**14a**	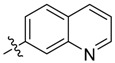	>100	100	>100	≥500	≥500	>250 *
Sulfamethoxazole		3.13	3.13	12.5	250	≥500	n.t.
Sulfaclozine		6.25	12.5	25	250	250	n.t.
Sulfaquinoxaline		3.13	12.5	50	125	250	n.t.
Benzenesulfonamide		>100	>100	>100	31.25	125	n.t.
**PZA** **		>100	>100	>100	≥500	≥500	>10^4^ [20]
**INH**		0.2	25	12.5	15.625	3.91	79 × 10^3^ [20]
**RFM**		n.t.	n.t.	n.t.	25	1.56	n.t.
**CPX**		n.t.	n.t.	n.t.	0.125	0.008	n.t.

* Measurement at higher concentrations was not possible due to precipitation of the tested compound in the cell culture medium. ** MIC value from testing at pH = 5.6 (acidic) is 6.25–12.5 μg/mL [2]. The value stated in the table is from testing at pH = 6.6 (neutral). *** Compound 4b is structurally identical to the sulfaclozine standard. n.t.—not tested. For structures of control drugs of the sulfonamide type refer to Figure 4.

**Table 2:** Experimental and calculated p*Ka* values of compounds **6b**, **9b**, and **13b**.

	Experimental		Calculated		
No.	Absorbance Method	Potentiometric Method	ChemAxon	MOE	ChemDraw
**6b**	6.95	7.21	6.21	8.30	NA
**9b**	6.32	6.38	6.29	8.30	NA
**13b**	5.63	5.75	6.01	8.30	NA

**Table 3:** p*Ka* values of title compounds predicted using the Chemicalize web application.

No.	p*Ka*	No.	p*Ka*	No.	p*Ka*	No.	p*Ka*	No.	p*Ka*
**1a**	6.11	**3b**	5.76	**6a**	6.30	**9a**	6.39	**12a**	6.11
**1b**	6.05	**4a**	6.61	**6b**	6.21	**9b**	6.29	**12b**	6.05
**2a**	6.16	**4b**	6.49	**7b**	6.03	**10a**	6.38	**13a**	6.07
**2b**	6.09	**5a**	6.10	**8a**	6.12	**11a**	6.16	**13b**	6.01
**3a**	5.80	**5b**	6.03	**8b**	6.05	**11b**	6.09	**14a**	6.07

**Table 4:** Comparison between the antimycobacterial activity against *Mtb* H37Rv expressed by the minimum inhibitory concentrations (MIC) in µg/mL.

Substituents		Antimycobacterial Activity MIC (µg/mL) against *Mtb* H37Rv		
R^1^	R^2^	Amide	Retro-amide	Sulfonamide
H	H	100	>100	>100 (1a)
H	4-OCH_3_	25	>100	>100 (6a)
6-Cl	H	>100	25	50 (1b)

R^1^—substitution on pyrazine, R^2^—substitution on benzene.

## Supplementary Materials

Biological assays; table of outcomes of target fishing; ^1^H-NMR and ^13^C-NMR spectra of compounds **6a** and **6b**.
