# Supplementary material for: Substituted N-(Pyrazin-2-yl)benzenesulfonamides; Synthesis, Anti-Infective Evaluation, Cytotoxicity, and In Silico Studies
Source: Molecules. 2019 Dec 29;25(1):138. doi: 10.3390/molecules25010138 (PMC6982817; doi:10.3390/molecules25010138)
Supplement: Supplementary file 1 [file molecules-25-00138-s001.pdf]

Supporting Information

Substituted *N*-(Pyrazin-2-yl)benzenesulfonamides; Synthesis, Anti-infective Evaluation, Cytotoxicity, and In Silico Studies.

Ghada Bouz [1,*], Martin Juhás [1], Lluis Pausas Otero [1], Cristina Paredes de la Red [1], Ondřej Janďourek [1], Klára Konečná [1], Pavla Paterová [2], Vladimír Kubíček [1], Jiří Janoušek [1], Martin Doležal [1] and Jan Zitko [1,*]

[1] Faculty of Pharmacy in Hradec Králové, Charles University, Heyrovského 1203, 50005 Hradec Králové, Czech Republic; juhasm@faf.cuni.cz (M.J.); lluis_pausas@hotmail.com (L.P.O.); cristinaparedesdelared@gmail.com (C.P.R.); JANDO6AA@faf.cuni.cz (O.J.); konecna@faf.cuni.cz (K.K.); kubicek@faf.cuni.cz (V.K.); janousj2@faf.cuni.cz (J.J.); dolezalm@faf.cuni.cz (M.D.)

[2] Department of Clinical Microbiology, University Hospital, Sokolská 581, 500 05 Hradec Králové, Czech Republic; pavla.paterova@fnhk.cz

* Correspondence: bouzg@faf.cuni.cz (G.B.); jan.zitko@faf.cuni.cz (J.Z.); Tel.: +420-495-067-275 (G.B.); +420-495-067-272 (J.Z.)

Biological assays

In Vitro Activity Evaluation Against Mycobacterium Tuberculosis, Mycobacterium Kansasii, and Mycobacterium Avium

A 96-well plate microdilution broth method was performed as described in Reference [1]. Tested strains Mycobacterium *tuberculosis* H37Rv CNCTC My 331/88 (ATCC 27294), *M. kansasii* CNCTC My 235/80 (ATCC 12478), and *M. avium ssp. avium* CNCTC My 80/72 (ATCC 15769) were obtained from the Czech National Collection of Type Cultures (CNCTC), National Institute of Public Health (Prague, Czech Republic). Middlebrook 7H9 broth of declared pH = 6.6 (Sigma-Aldrich) enriched with 0.4% of glycerol (Sigma-Aldrich) and 10% of OADC growth supplement (oleic acid, albumin, dextrose, catalase; Himedia, Mumbai, India) was used for cultivation. The tested compounds were dissolved and diluted in DMSO and mixed with broth (25 μL of DMSO solution in 4.475 mL of broth) and then placed (100 μL) into microplate wells. Mycobacterial inocula were suspended in isotonic saline solution and the density was adjusted to 0.5–1.0 according to the McFarland scale. These suspensions were diluted by 10 $^{-1}$ and used to inoculate the testing wells, by adding 100 μL of mycobacterial suspension per well. The final concentrations of tested compounds in wells were 100, 50, 25, 12.5, 6.25, 3.13, and 1.56 μg/mL. INH was used as a positive control (inhibition of growth). The negative control (visible growth) consisted of broth plus mycobacterial suspension plus DMSO (purity of broth). A total of 30 μL of Alamar Blue working solution (1:1 mixture of 0.01% resazurin sodium salt (aq. sol.) and 10% Tween 80) was added after five days of incubation. Results were determined after 24 h of incubation. The MIC (in μg/mL) was determined as the lowest concentration that prevented the blue to pink color change. The MIC values of INH were 6.25–12.5 μg/mL against *M. avium*, 3.13–12.5 μg/mL against *M. kansasii*, and 0.1–0.2 μg/mL against *M. tuberculosis* H37Rv. All experiments were conducted in duplicates.

In Vitro Activity Evaluation Against Mycobacterium Smegmatis and Mycobacterium Aurum

The antimycobacterial assay was performed with fast-growing *Mycobacterium smegmatis* DSM 43465 (ATCC 607) and *Mycobacterium aurum* DSM 43999 (ATCC 23366) from the German Collection of Microorganisms and Cell Cultures (Braunschweig, Germany). The technique used for activity determination was the microdilution broth panel method using 96-well microtitration plates. Culturing medium was Middlebrook 7H9 broth (Sigma-Aldrich) enriched with 0.4% of glycerol (Sigma-Aldrich) and 10% of Middlebrook OADC growth supplement (Himedia, Mumbai, India). Mycobacterial strains were cultured on Middlebrook 7H10 agar and the suspensions were prepared in Middlebrook 7H9

broth. The final density was adjusted to a value ranging from 0.5 to 1.0, according to the McFarland scale and diluted in ratio 1:20 with broth. Tested compounds were dissolved in DMSO (Sigma-Aldrich), then Middlebrook broth was added to obtain a concentration of 2000 μg/mL. Standards used for activity determination were isoniazid (INH), rifampicin (RIF), and ciprofloxacin (CPX) (Sigma-Aldrich). The final concentrations were reached using a binary dilution and addition of mycobacterial suspension. The concentrations were set as 500, 250, 125, 62.5, 31.25, 15.625, 7.81, and 3.91 μg/mL except for the standards of rifampicin, where the final concentrations were 12.5, 6.25, 3.125, 1.56, 0.78, 0.39, 0.195, and 0.098 μg/mL, and ciprofloxacin, where the final concentrations were 1, 0.5, 0.25, 0.125, 0.0625, 0.0313, 0.0156, and 0.0078 μg/mL.

The final concentration of DMSO did not exceed 2.5% (v/v) and it did not affect the growth of *M. smegmatis* nor *M. aurum*. Positive (broth, DMSO, bacteria) and negative (broth, DMSO) controls were included. The plates were sealed with polyester adhesive film and incubated in the dark at 37 °C without agitation. The addition of a 0.01% solution of resazurin sodium salt followed after 48 hours of incubation for *M. smegmatis*, and after 72 hours of incubation for *M. aurum*. The stain was prepared by dissolving resazurin sodium salt (Sigma-Aldrich) in deionized water to get a 0.02% solution. Then 10% aqueous solution of Tween 80 (Sigma-Aldrich) was prepared. Both liquids were mixed up making use of the same volumes and filtered through a syringe membrane filter. Microtitration panels were then incubated for a further 2.5 hours for the determination of activity against *M. smegmatis*, and 4 hours for *M. aurum*. The antimycobacterial activity was expressed as the MIC and the value was read based on the stain colour change (blue colour – no growth; pink colour – growth). The MIC values for the standards were in the ranges 7.81–15.625 μg/mL for INH, 12.5–25 μg/mL for RIF, and 0.0625–0.125 μg/mL for CPX against *M. Smegmatis*. Meanwhile, the ranges were 1.95–3.91 μg/mL for INH, 0.78–1.56 μg/mL for RIF, and 0.00781–0.01563 μg/mL for CPX against *M. aurum*, respectively. All experiments were conducted in duplicates.

In Vitro Antibacterial Activity Evaluation

Microdilution broth method as described in Reference [2]. We tested the following strains from the Czech Collection of Microorganisms (CCM, Brno, Czech Republic): *Staphylococcus aureus* CCM 4223 (ATCC 29213), *Staphylococcus aureus* methicillin-resistant CCM 4750 (ATCC 43300), *Enterococcus faecalis* CCM 4224 (ATCC 29212), *Escherichia coli* CCM 3954 (ATCC 25922), and *Pseudomonas aeruginosa* CCM 3955 (ATCC 27853). We obtained the following clinical isolates from the Department of Clinical Microbiology, University Hospital in Hradec Králové, Czech Republic: *Staphylococcus epidermidis* 112-2016, *Klebsiella pneumoniae* 64-2016, and *Serratia marcescens* 62-2016. All strains were subcultured in Mueller-Hinton agar (MHA) (Difco/Becton Dickinson, Detroit, MI, USA) at 35 °C and maintained in the same medium at 4 °C. The compounds were dissolved in DMSO, and the antibacterial activity was determined in cation adjusted Mueller–Hinton liquid broth (Difco/Becton Dickinson) buffered to pH 7.0. Positive controls consisted of the test microbe, while negative controls consisted of the cultivation medium and DMSO. The final concentration of DMSO in the testing medium did not exceed 1% (v/v) of the total solution composition. MIC was determined after 24 and 48 h of static incubation at 35 °C by visual inspection or using Alamar Blue dye. The standards were gentamicin [MIC against *Staphylococcus aureus* 1 μg/mL (48 h); *Staphylococcus aureus* methicillin resistant 16–32 μg/mL (48 h); *Enterococcus faecalis* 8 μg/mL (48 h); *Escherichia coli* 1–2 μg/mL (48 h); *Pseudomonas aeruginosa* 0.5 μg/mL (48 h); *Staphylococcus epidermidis* >8 μg/mL (48 h); *Klebsiella pneumonia* >8 μg/mL (48 h); *Serratia marcescens* 2 μg/mL (48 h)] and ciprofloxacin [MIC against *Staphylococcus aureus* 0.128–0.256 μg/mL (48 h); *Staphylococcus aureus* methicillin resistant 0.128 μg/mL (48 h); *Enterococcus faecalis* 0.512 μg/mL (48 h); *Escherichia coli* 0.008 μg/mL (48 h); *Pseudomonas aeruginosa* 0.128 μg/mL (48 h); *Staphylococcus epidermidis* >1.024 μg/mL (48 h); *Klebsiella pneumonia* >1.024 μg/mL (48 h); *Serratia marcescens* 0.256 μg/mL (48 h)]. All experiments were conducted in duplicates. For the results to be valid, the difference in MIC for one compound determined from two parallel measurements must not be greater than one step on the dilution scale.

In Vitro Antifungal Activity Evaluation

Microdilution broth method as described in References [3,4]. We tested the following strains from the Czech Collection of Microorganisms (CCM): Candida albicans CCM 8320 (ATCC 24433), C. krusei CCM 8271 (ATCC 6258), C. parapsilosis CCM 8260 (ATCC 22019), C. tropicalis CCM 8264 (ATCC 750), Aspergillus flavus CCM 8363, Lichtheimia corymbifera CCM 8077, and Trichophyton interdigitale CCM 8377 (ATCC 9533); and from the American Type Collection Cultures (ATCC, Mannasas, VA, USA), Aspergillus fumigatus ATCC 204305. The compounds were dissolved in DMSO and diluted in a twofold manner with an RPMI 1640 medium, with glutamine and 2% glucose, buffered to pH 7.0 with MOPS (3-morpholinopropane-1-sulfonic acid). The final concentration of DMSO in the testing medium did not exceed 1% (v/v) of the total solution composition. Static incubation was performed in the dark and in a humid atmosphere, at 35 °C, for 24 and 48 h (and 72 and 120 h for the Trichophyton interdigitale, respectively). Positive controls consisted of the test microbe while negative controls consisted of the cultivation medium and DMSO. The MIC was inspected visually or by making use of Alamar Blue indication. The standards were amphotericin B [MIC against Candida albicans 0.5 μg/mL (48 h); C. krusei 1 μg/mL (48 h); C. parapsilosis 0.5 μg/mL (48 h); C. tropicalis 1 μg/mL (48 h); Aspergillus flavus 8 μg/mL (48 h); Lichtheimia corymbifera 0.5 μg/mL (48 h); Trichophyton interdigitale 2 μg/mL (72 h); Aspergillus fumigatus 1 μg/mL (48 h)] and voriconazole [MIC against Candida albicans >16 μg/mL (48 h); C. krusei 0.5 μg/mL (48 h); C. parapsilosis 8 μg/mL (48 h); C. tropicalis >16 μg/mL (48 h); Aspergillus flavus >16 μg/mL (48 h); Lichtheimia corymbifera >16 μg/mL (48 h); Trichophyton interdigitale >16 μg/mL (72 h); Aspergillus fumigatus 1 μg/mL (48 h)]. All experiments were conducted in duplicates. For the results to be valid, the difference in MIC for one compound determined from two parallel measurements must not be greater than one step on the dilution scale.

Cytotoxicity Determination

Human hepatocellular liver carcinoma cell line HepG2 (passage 9–12) purchased from the Health Protection Agency Culture Collections (ECACC, Salisbury, UK). They were cultured in MEM (minimum essentials Eagle medium) (Sigma-Aldrich, St. Louis, USA) supplemented with 10% fetal bovine serum (PAA), 1% L-Glutamine solution (Sigma-Aldrich), and non-essential amino acid solution (Sigma-Aldrich) in a humidified atmosphere containing 5% CO_2 at 37°C. For subculturing, the cells were harvested after trypsin/EDTA (Sigma-Aldrich) treatment at 37°C. To evaluate cytotoxicity, we used the cells treated with the tested substances as experimental groups, whereas untreated HepG2 cells served as controls. The cells were seeded at a density of 10,000 cells per well in a 96-well plate. On the following day, the cells were treated with each of the tested substances dissolved in DMSO. The tested substances were prepared at different incubation concentrations (Table 2) in triplicates according to their solubility. Simultaneously, the controls representing 100% cell viability, 0% cell viability (the cells treated with 10% DMSO), no cell control, and vehiculum controls, were also prepared in triplicates. After 24 h incubation in a humidified atmosphere containing 5% CO_2 at 37%, we added the reagent from the kit CellTiter 96 Aqueous One Solution Cell Proliferation Assay (CellTiter 96; PROMEGA, Fitchburg, USA). After 2 h incubation at 37%, the absorbance of samples was recorded at 490 nm (TECAN, Infinita M200, Austria). A nonlinear regression calculated a standard toxicological parameter IC50 from a semilogarithmic plot of incubation concentration versus the percentage of absorbance relative to untreated controls using the GraphPad Prism 7 software.

Table S1. Outcomes of target fishing.

Target	Organism	PDB ID	Original ligand	Ligand ID (PDB)/Name	Note
Dihydropteroate synthase	*Bacillus anthracis*	3tye	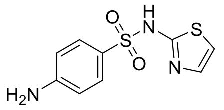	YTZ (sulfathiazole)	Docked
Dihydropteroate synthase	*Yersinia pestis*	3tzf	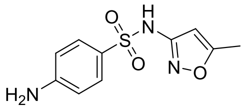	08D (sulfamethoxazole)	Docked to PDB ID 3tye[1]
GTPase KRas	*Homo sapiens*	4epx	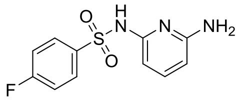	0QR	Docked, results not presented[2]
Sepiapterin reductase	*Homo sapiens*	4hwk	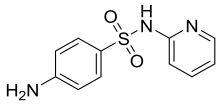	SFY (sulfapyridine)	Not investigated
Sepiapterin reductase	*Homo sapiens*	4j7u	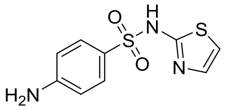	YTZ (sulfathiazole)	Not investigated
Serine/threonine-protein kinase B-raf	*Homo sapiens*	4xv9	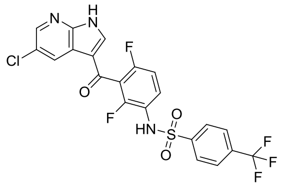	1OO	Not investigated[3]
β-Lactamase VIM-2	*Pseudomonas aeruginosa*	5mxq	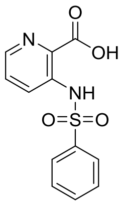	U8K	Not investigated
Matrix metalloproteinase MMP-8	*Homo sapiens*	5h8x	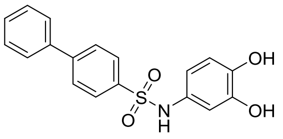	5XT	Docked
DNA damage-binding protein 1	*Homo sapiens*	6pai	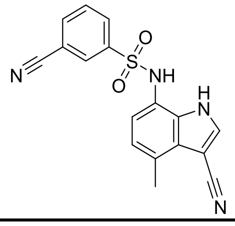	O6M	Not investigated

[1] results have been presented in part *2.4 Biological activity.*

[2] no particularly interesting poses were observed.

[3] patented as inhibitors of B-raf kinase [5].

1. H-NMR of compound 6a

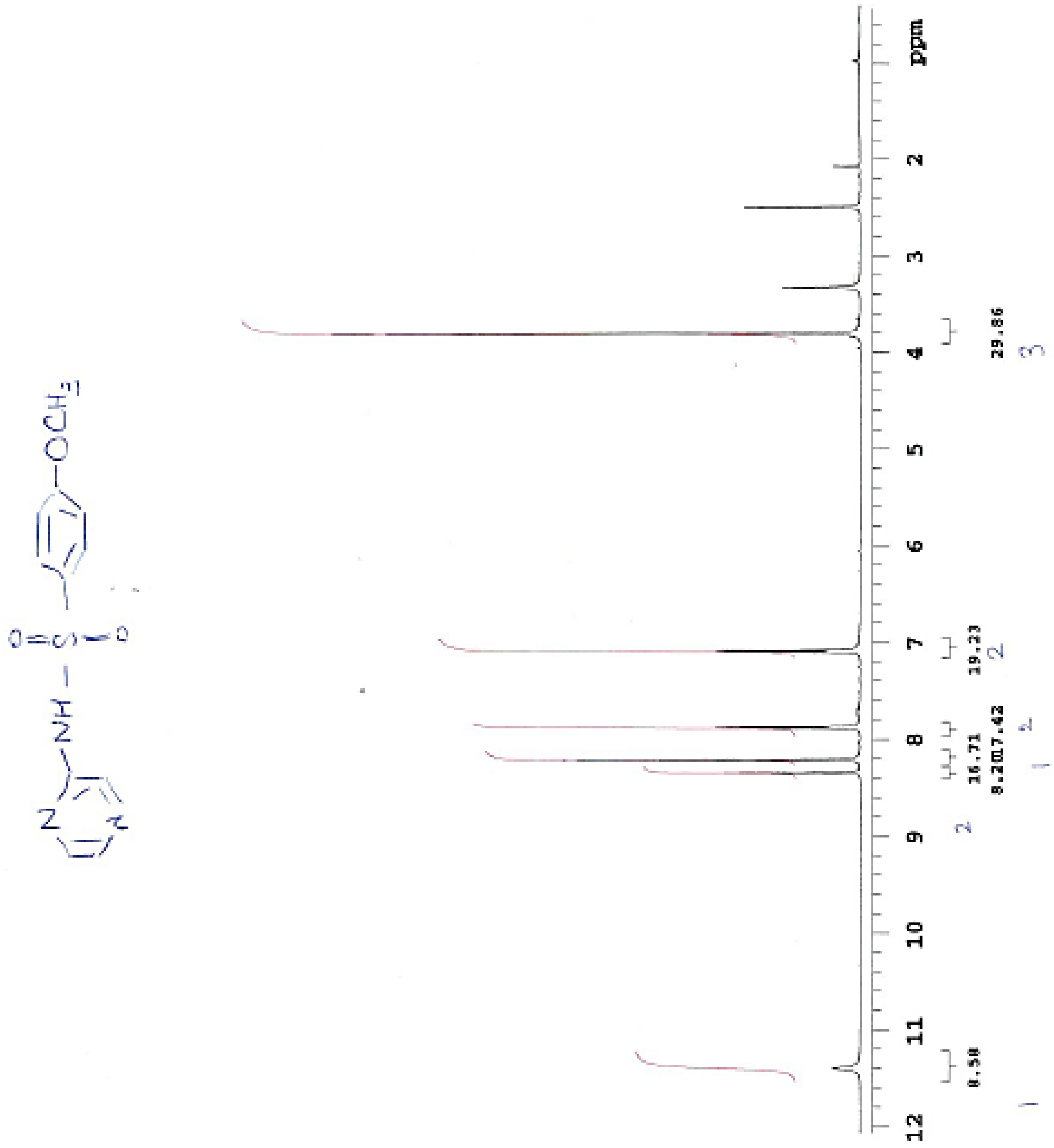

13. C-NMR of compound 6a

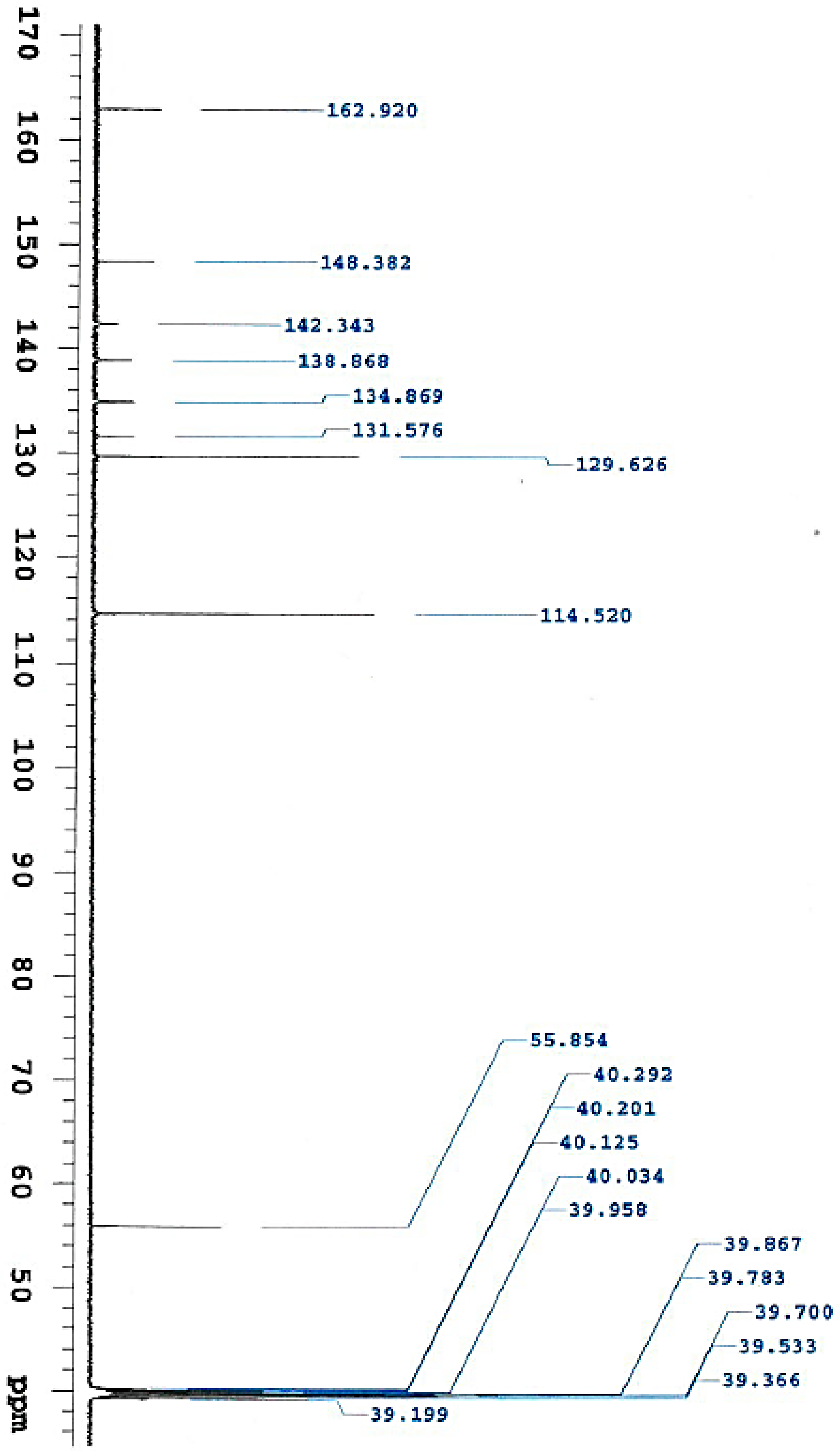

[1.] **H-NMR of compound 6b**

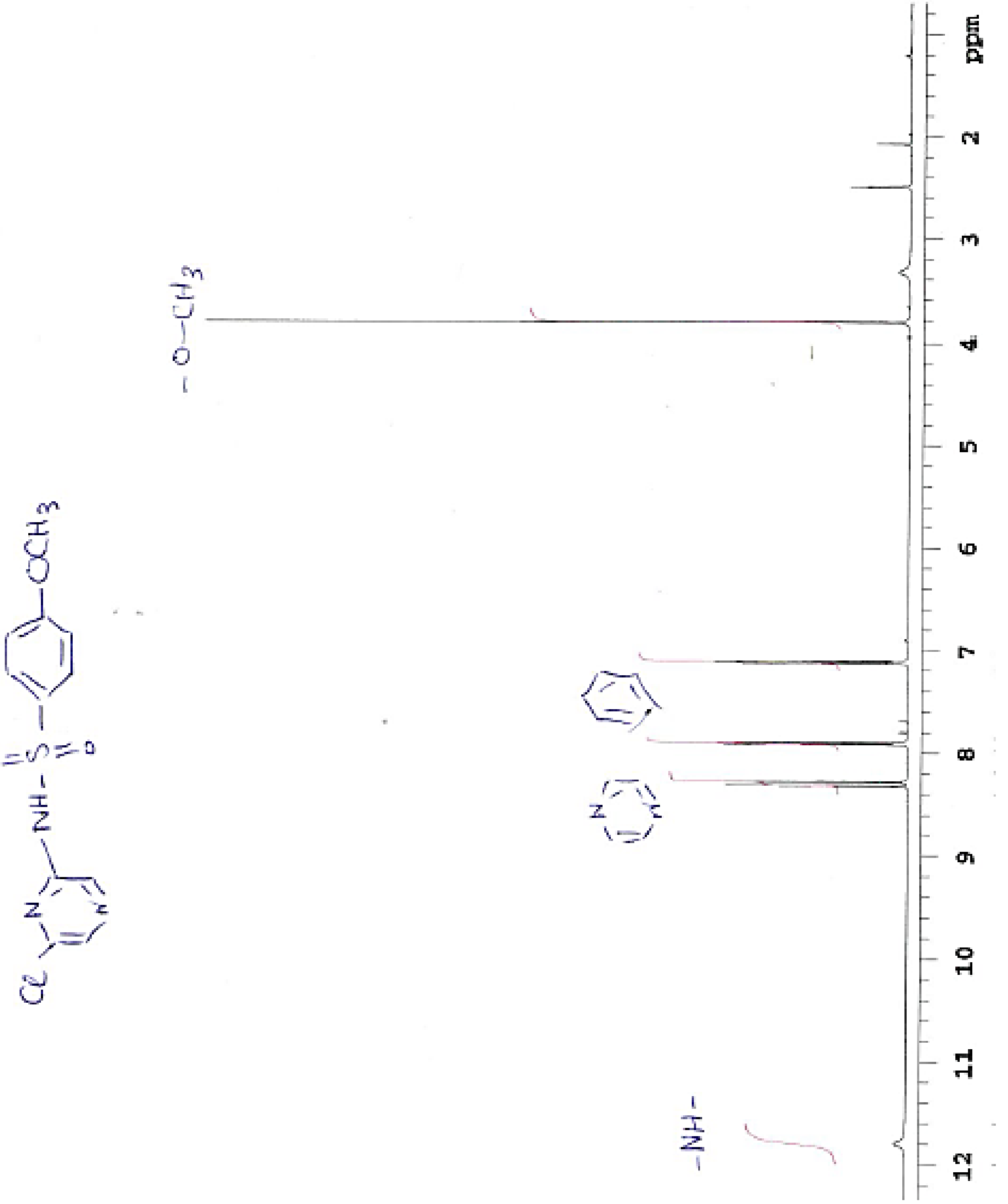

[13] C-NMR of compound 6b

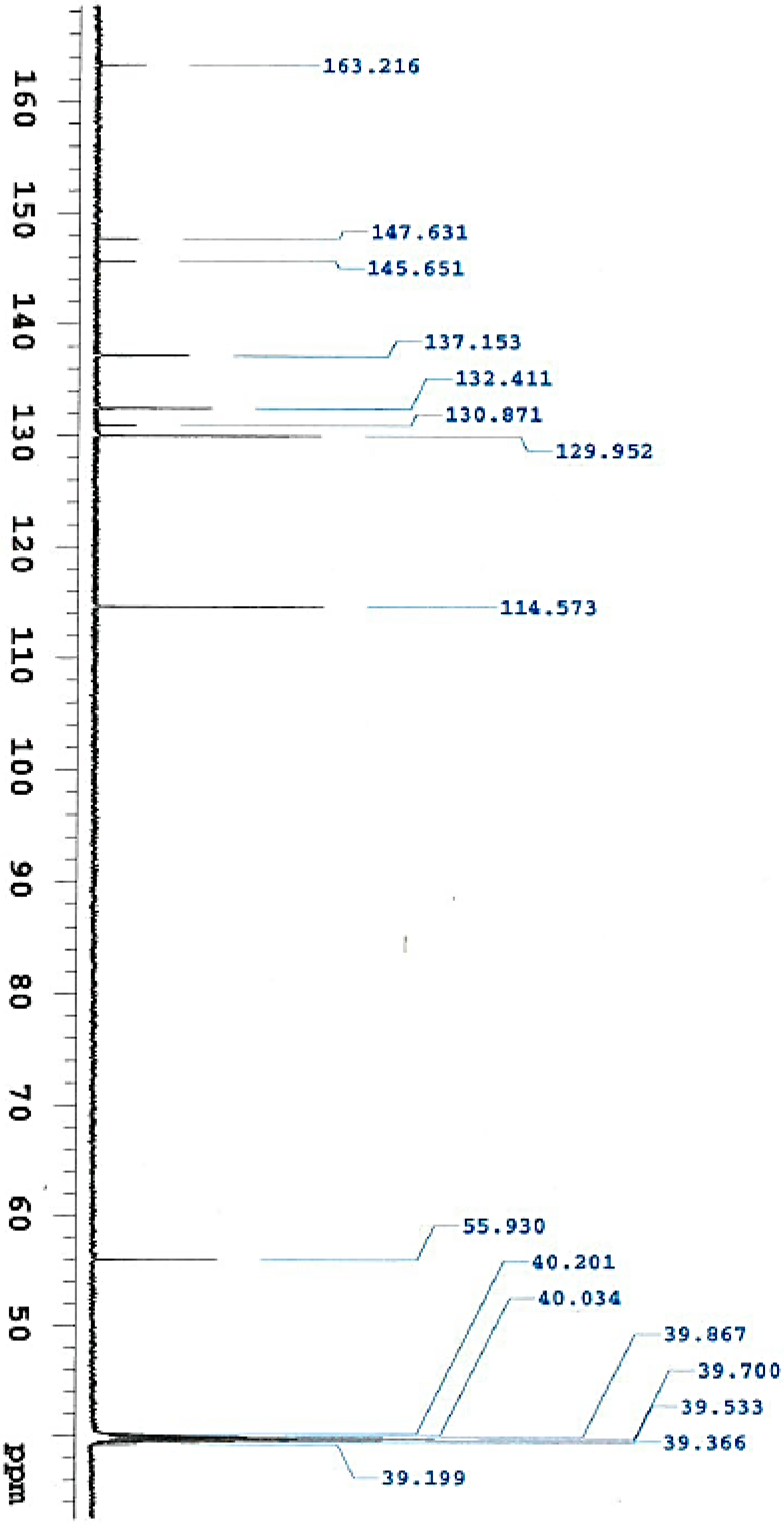

References

1. Franzblau, S.G.; Witzig, R.S.; McLaughlin, J.C.; Torres, P.; Madico, G.; Hernandez, A.; Degnan, M.T.; Cook, M.B.; Quenzer, V.K.; Ferguson, R.M.; et al. Rapid, low-technology MIC determination with clinical Mycobacterium tuberculosis isolates by using the microplate Alamar Blue assay. *J. Clin. Microbiol.* **1998,** *36*, 362–366.
2. EUCAST DISCUSSION DOCUMENT E.Dis 5.1. Determination of Minimum Inhibitory Concentrations (MICs) of Antibacterial Agents by Broth Dilution. *Clin. Microbiol. Infect.* **2003**, *9*, 1–7.
3. EUCAST DEFINITIVE DOCUMENT E.DEF 9.3.1. Method for the Determination of Broth Dilution Minimum Inhibitory Concentrations of Antifungal Agents for Conidia Forming Moulds; EUCAST: Växjö, Sweden, 2017; pp. 1–23.
4. EUCAST DEFINITIVE DOCUMENT E.DEF 7.3.1. Method for the Determination of Broth Dilution Minimum Inhibitory Concentrations of Antifungal Agents for Yeasts; EUCAST: Växjö, Sweden, 2017; pp. 1–21.
5. Bamford, M.; Dean, D.; Naylor, A.; Takle, A.; Wilson, D. Nitrogen-Containing Heterocyclic Compounds as Inhibitors of B-Raf Kinase. U.S. Patent 7297693B2, 2007.
